# Signal Propagation in Protein Interaction Network during Colorectal Cancer Progression

**DOI:** 10.1155/2013/287019

**Published:** 2013-03-20

**Authors:** Yang Jiang, Tao Huang, Lei Chen, Yu-Fei Gao, Yudong Cai, Kuo-Chen Chou

**Affiliations:** ^1^Department of Surgery, China-Japan Union Hospital of Jilin University, Changchun 130033, China; ^2^Department of Genetics and Genomic Sciences, Mount Sinai School of Medicine, New York, NY 10029, USA; ^3^College of Information Engineering, Shanghai Maritime University, Shanghai 201306, China; ^4^Institute of Systems Biology, Shanghai University, Shanghai 200444, China; ^5^King Abdulaziz University, Jeddah, Saudi Arabia; ^6^Gordon Life Science Institute, 53 South Cottage Road, Belmont, MA 02478, USA

## Abstract

Colorectal cancer is generally categorized into the following four stages according to its development or serious degree: Dukes A, B, C, and D. Since different stage of colorectal cancer actually corresponds to different activated region of the network, the transition of different network states may reflect its pathological changes. In view of this, we compared the gene expressions among the colorectal cancer patients in the aforementioned four stages and obtained the early and late stage biomarkers, respectively. Subsequently, the two kinds of biomarkers were both mapped onto the protein interaction network. If an early biomarker and a late biomarker were close in the network and also if their expression levels were correlated in the Dukes B and C patients, then a signal propagation path from the early stage biomarker to the late one was identified. Many transition genes in the signal propagation paths were involved with the signal transduction, cell communication, and cellular process regulation. Some transition hubs were known as colorectal cancer genes. The findings reported here may provide useful insights for revealing the mechanism of colorectal cancer progression at the cellular systems biology level.

## 1. Background

Cancer is a complex system disease [1]. The complexity reflects in many ways. First, it is a network disease that involves the changes of many genes and these genes are connected in a certain way. Second, the disease network is evolving all the time during the progression. Some efforts have been made to understand such dynamic network [2–6]. 

As the third most common cancer worldwide [7], colorectal cancer develops via a progressive accumulation of genetic mutations and pathway dysfunctions [6]. It has the following four stages from early to late [8]: Dukes A, B, C, and D. In the stage of Dukes A, the cancer is only limited to the innermost layer. In Dukes B stage, the cancer has grown through the muscle layer. In Dukes C stage, the cancer has spread to the lymph nodes nearby. In Dukes D stage, the cancer is widely spread. The stage of Dukes D is the most advanced stage of colorectal cancer. Understanding the underlying molecular mechanisms of the pathological changes in colorectal cancer progression will facilitate the development of therapeutic treatments.

In the study of prion disease, it was found that during different stages of the disease, different regions of the network were activated and they formed a clear disease aggravation pattern on the network [2]. However, it is still not clear how one activated region is connected with another and how they can transit into one another. 

To investigate the transition processes of different network states, we analyzed the gene expression profiles of 290 colorectal cancer patients, who were at different stages of Dukes A, B, C, and D. Using the Maximum Relevance and Minimum Redundancy (mRMR) [9] and Incremental Feature Selection (IFS) methods [10, 11] to compare the gene expressions among the patients of Dukes A, B, C, and D stages, we obtained 158 early stage biomarkers and 284 late stage biomarkers, respectively. Subsequently, the early stage biomarkers and the late stage biomarkers were mapped onto the protein interaction network. If the early stage biomarker and the late stage biomarker were close to each other in the network, and also their expression levels were correlated with the patients of the Dukes B and C stages, then we assume that a signal propagation path may exist from the early stage biomarker to the late stage biomarker. Thus, by screening all the possible signal propagation paths from the early stage biomarkers to the late stage biomarkers, we have identified 632 signal propagation paths that contained 473 transition genes. 

According to the Gene Ontology (GO) [12] enrichment analysis, many of the transition genes that transmitted the disease signal from the early stage biomarkers to the late stage biomarkers were involved into the signal transduction, cell communication, and cellular process regulation. Some transition hub genes were known colorectal cancer genes. They helped the transduction of the disease signal and the aggravation of colorectal cancer.

One signal propagation path from early stage biomarker MAVS to late stage biomarker GFPT1 was shown as an example. MAVS is an important immune protein and signaling protein in mitochondria [13–15] and GFPT1 is a rate-limiting enzyme of metabolism [16, 17]. It was suggested through our signal propagation analysis that MAVS responded to colorectal cancer in the early stage and then transmitted the disease signal to GFPT1 whose dysfunction further accelerated the colorectal cancer patients into late stage. This kind of in-depth analysis on the signal propagation path may provide useful insights into, or enrich, the understanding of the mechanism of colorectal cancer at the cellular or system biology level.

## 2. Methods

### 2.1. Benchmark Dataset

We downloaded the expression profiles of 19,621 genes in 290 colorectal cancer patients [18] from Gene Expression Omnibus (GEO) under accession number GSE14333. Of the 290 colorectal cancer patients, 44 were Dukes stage A, 94 Dukes stage B, 91 Dukes stage C, and 61 Dukes stage D. From Dukes A stage to Dukes D, the colorectal cancer gets more and more severe.

The protein interaction network we used was STRING v9.0 (http://string-db.org/) [19]. Each protein interaction in STRING has a confidence score, varying from 0.150 to 1. The confidence score is calculated by integrating the functional associations from genomic context, experiments, conserved coexpression, and previous knowledge with Bayesian method [19]. Suppose the interaction confidence score is denoted by *𝕀*_score_, it follows according to the original definition

(1) $$\begin{array}{c} {𝕀}_{\mathrm{rank}}=\{\begin{array}{cc} \text{low}\;\text{confidence}, & \text{if}\;I_{\text{score}}>0.150\\ \text{mediam}\;\text{confidence}, & \text{if}\;I_{\text{score}}>0.400\\ \text{high}\;\text{confidence}, & \text{if}\;I_{\text{score}}>0.700\\ \text{highest}\;\text{confidence}, & \text{if}\;I_{\text{score}}>0.900, \end{array} \end{array}$$

where *𝕀*_rank⁡_ represents the rank of protein interaction.

### 2.2. The Diagram of Signal Propagation Analysis during Cancer Progression

In studying or analyzing complex biological systems, it is quite helpful to introduce graphs or diagrams since they can provide an overall view or intuitive insights for the systems investigated, as demonstrated by a series of studies on various important biological topics (see, e.g., [20–29]). In this study, we first constructed a graph *𝔾* with the PPI data from STRING. In the graph, an edge was assigned for each pair of proteins if they were in interaction with each other. There were 1375295 interaction edges among 15240 proteins. The “intimate degree” between two interacting proteins was defined by

(2) $$\begin{array}{c} {𝕀}_{\text{intimate}}=1000\times \left(1-{𝕀}_{\text{score}}\right), \end{array}$$

where *𝕀*_score_ is the confidence score between two proteins concerned [19]. Thus, the higher the interaction confidence score between two proteins is, the closer their “interactive distance,” and hence more intimate between them.

**Figure 1:**
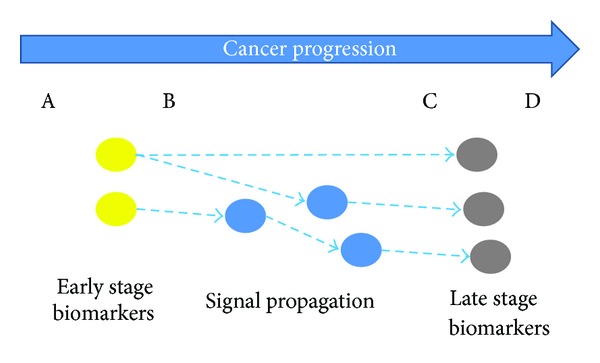
The diagram of signal propagation analysis during cancer progression. The blue arrow represents cancer progression. The colorectal cancer has four stages: Dukes A, B, C, and D. From A to D, the cancer gets worse and worse. Yellow nodes and grey nodes represent the biomarkers in the early and late stage, respectively. The goal of signal propagation analysis is to understand the transition from early stage biomarkers to late stage biomarkers by analyzing the signal propagation in the protein interaction network.

Shown in Figure 1 is an illustration for analyzing the signal propagation during the cancer progression. The colorectal cancer has four stages: Dukes A, B, C, and D. From Dukes A to Dukes D, the cancer gets worse and worse. The blue arrow represents the cancer progression. Below, we are to identify the biomarkers in the early stage (yellow nodes) and biomarkers in the late stage (grey nodes). Subsequently, we try to understand the transition from early stage biomarkers to late stage biomarkers by analyzing the signal propagation in the protein interaction network. This kind of analysis may provide useful insights for us to in-depth understand how the signal is propagated through the network.

### 2.3. Identification of Biomarkers in the Early and Late Stage

The following methods were used to identify the genes between different Dukes stages. First, the Maximum Relevance and Minimum Redundancy (mRMR) [9] method was applied to select the genes that has both maximum relevance with the cancer stages and minimum redundancy to each other. The mRMR program was downloaded from http://penglab.janelia.org/proj/mRMR/. Second, the mRMR ranked genes were optimized with the Incremental Feature Selection (IFS) method [8, 30–35]. During the IFS operation, the accuracies of all possible top gene sets were calculated and the gene set that had the highest prediction accuracy was chosen as the optimal gene set, that is, the biomarkers. The accuracy was examined by the jackknife test, also known as Leave-One-Out Cross Validation (LOOCV) [36–39] and the prediction model was Nearest Neighbor Algorithm (NNA) [40]. The prediction accuracy was defined as the number of correctly predicted samples divided by the number of total samples. 

The early stage biomarkers were selected from the Dukes A patients and Dukes B patients with mRMR and IFS methods. The late stage biomarkers were selected from the Dukes C patients and Dukes D patients.

### 2.4. The Transition from the Early Stage Biomarkers to the Late Stage Biomarkers

The early stage biomarkers and late stage biomarkers were mapped onto weighted protein interaction network graph *𝔾*. We identified the shortest paths between them using Dijkstra's algorithm [41–43]. The path length was the sum of edge weights through which the path passed. If the path length was smaller than 1000 × (1 − 0.700) = 300, it had high confidence to happen.

Meanwhile, we also tested the correlation between early stage biomarkers and late stage biomarkers in Dukes B patients and Dukes C patients. The Pearson correlation test *P* values were adjusted with false discovery rate (FDR) [44]. The cutoff of Pearson correlation test FDR was set to 0.001.

Included were those transitions that had the length shorter than 300 and the correlation test FDR smaller than 0.001. The shortest paths from the early stage biomarkers to the late stage biomarkers in the protein interaction network were deemed as the signal propagation paths for the transition.

### 2.5. Statistical Significance of Signal Propagation Path Identification

To evaluate the statistical significance of the identified signal propagation paths, we estimated the FDR of the signal propagation path based on the permutation [45]. We permuted the gene symbols in protein interaction network and gene expression profiles by 20,000 times. For each of the permutations, we calculated the length of the shortest path based on the weighted protein interaction network and the Pearson correlation test *P* value adjusted with the FDR method based on the gene expression profiles. The FDR of the signal propagation path was defined as

(3) $$\begin{array}{c} {\text{FDR}}_{\text{signal-path}}=\frac{N_{1}}{N_{2}}, \end{array}$$

where *N*_1_ was the number of permutations in which the permuted shortest path length is shorter than the actual shortest path length and the permuted Pearson correlation test FDR is smaller than the actual Pearson correlation test FDR, while *N*_2_ the total number of permutations which was 20,000 in this study.

### 2.6. The Transition Hubs in the Signal Propagation Paths

For each of the transition genes, we calculated the number of shortest paths that crossed it. Those genes that were crossed by more signal propagation paths were deemed more important transition hubs.

## 3. Results

### 3.1. Early and Late Stage Biomarkers

**Figure 2:**
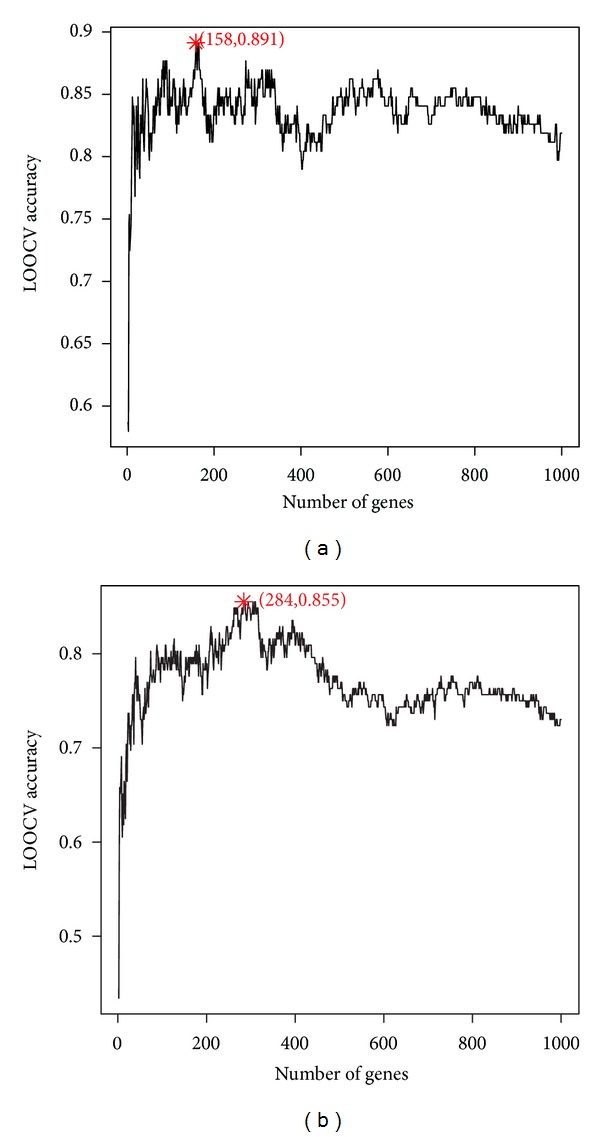
The IFS curves of early stage biomarkers and late stage biomarker. (a) The IFS curves of early stage biomarker selection. The highest accuracy was 0.891 with 158 genes which were the early stage biomarkers. (b) The IFS curves of late stage biomarker selection. The highest accuracy was 0.855 with 284 genes which were the late stage biomarkers.

By selecting discriminative genes between the Dukes A patients and the Dukes B patients with mRMR and IFS methods, we identified the early stage biomarkers. Similarly, we obtained the late stage biomarkers from the Dukes C patients and the Dukes D patients. The IFS curves of early and late stage biomarker selection were shown in Figures 2(a) and 2(b), respectively. In Figure 2(a), the highest accuracy was 0.891 with 158 genes of the early stage biomarkers. In Figure 2(b), the highest accuracy was 0.855 with 284 genes of the early stage biomarkers. The 158 early stage biomarkers and 284 late stage biomarkers can be found in Supplemental Tables S1 and S2, available online at http://dx.doi.org/10.1155/2013/287019 respectively.

### 3.2. Comparison of Early and Late Stage Biomarkers

Now let us compare the early stage biomarkers with the late stage ones. It was observed between the two kinds of biomarkers there was only one gene, RNF4, in common. The expected number of overlap genes should be 2.29 and the odds ratio was 0.432. In other words, there was less overlap than expected. It was reported that in different stages of disease, different regions of the biological network are activated [2] and the dynamics of the biological network reflects the histopathology and clinical changes [6, 46]. The shifting from the activated region of early stage biomarkers to the activated region of late stage biomarkers in the biological network explains the under overlap between the early and late stage biomarkers, which may also help understand the colorectal cancer progression. In the following section, we are to study the transition processes in which the early stage biomarkers propagate the disease-aggravating signal to the late stage biomarkers, triggering the patients to develop into the most severe condition.

### 3.3. From Early Stage Biomarkers to Late Stage Biomarkers: The Transition

**Figure 3:**
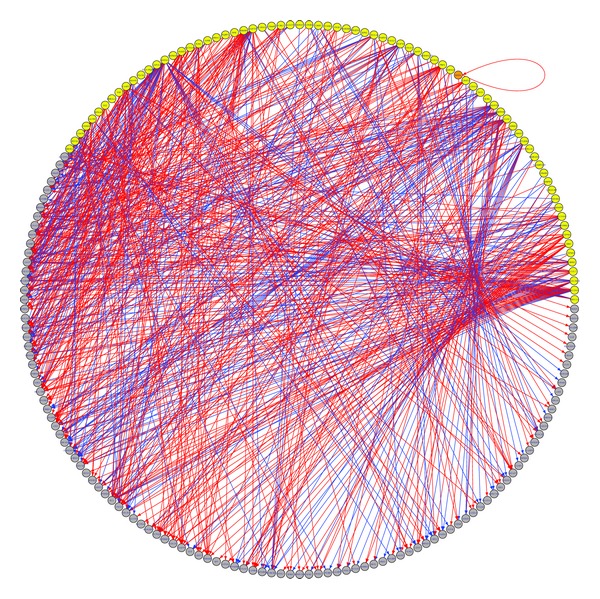
The transition network from early stage biomarkers to late stage biomarkers. The yellow and grey nodes were early and late stage biomarkers, respectively. The orange node, RNF4, was both early and late stage biomarker. The red and blue edges indicated that the early and late stage biomarkers were positively and negatively correlated.

There were 136 early stage biomarkers and 230 late stage biomarkers that could be mapped onto the STRING network. The number of all possible combination pairs between the early and late stage biomarkers was 136 × 230 = 31,280, for each of which we calculated their shortest path length that was the sum of the edge weights in the shortest path. Furthermore, we calculated the Pearson correlation test FDR between them in Dukes B patients and Dukes C patients. Two criteria were applied to get the signal propagation path from early stage biomarkers to late stage biomarkers: the path length should be shorter than 300 and the correlation test FDR should be smaller than 0.001. There were 632 such signal propagation paths, as given in Table S3. Such 632 signal propagation paths linked 76 early stage biomarkers and 109 late stage biomarkers. Shown in Figure 3 are the transition networks from early stage biomarkers to late stage biomarkers. 

Meanwhile, the values of FDR for the identified signal propagation paths were also calculated by first permuting the gene symbols in the protein interaction network and gene expression profiles and then comparing the permuted shortest path length and Pearson correlation FDR with the actual ones. Based on the results of the 20,000 permutations, the statistical significance of each identified signal propagation path was evaluated. It was found that all the 632 identified signal propagation paths were with FDR less than 0.05 and 81.3% of them had FDR less than 0.01.

### 3.4. The Transition Hubs in Signal Propagation

The 632 signal propagation paths crossed 473 genes. We ranked each of the 473 transition genes based on the number of signal propagation paths that had crossed it. The genes crossed by more signal propagation paths were regarded as more important transition hubs. The detailed results of the 473 transition genes as well as the numbers of signal propagation paths that had crossed them can be found in Table S4. The top three transition hubs were TP53 (tumor protein 53), CTNNB1 (cadherin-associated protein, beta 1), and EP300 (E1A binding protein p300). Interestingly, two of them, TP53 and EP300, were colorectal cancer genes, fully consistent with the reports in the Online Mendelian Inheritance in Man [47] (OMIM, http://omim.org/entry/114500). 

## 4. Discussion

### 4.1. The Biological Functions of Early Stage Biomarkers, Late Stage Biomarkers, and Transition Genes

We used GATHER [48] (http://gather.genome.duke.edu/) to investigate the biological functions of the 158 early stage biomarkers, the 284 late stage biomarkers, and the 473 transition genes. The Gene Ontology (GO) enrichment results thus obtained are shown in Tables 1, 2, and 3, respectively. Since the 473 transition genes were enriched into too many GO terms, only the five enriched GO terms with the highest Bayes factor [49] were shown in Table 3. It is instructive to point out that the late stage biomarkers had more enriched GO terms than the early stage biomarkers. Also, the late stage biomarkers were more enriched in the common GO terms than the early stage biomarkers, such as “GO:0009607: response to biotic stimulus,” “GO:0006952: defense response,” and “GO:0006955: immune response.” The roles of defense response and immune response in colorectal cancer [50, 51] have been widely studied. Many of the transition genes were involved in the signal transduction, cell communication, and cellular process regulation. These kinds of functions played important roles in transducing the disease signal and aggravating the colorectal cancer.

### 4.2. The Overlapped Gene between Early Stage Biomarkers and Late Stage Biomarkers

One overlapped gene, RNF4 (RING finger protein 4), was observed between the early stage biomarkers and the late stage biomarkers. As reported in [52], RNF4 was a patented biomarker gene of colorectal cancer. Also, as reported in [53], downregulation of RNF4 was related to the colorectal cancer risk (http://www.wipo.int/patentscope/search/en/WO2010033371).

Since RNF4 plays a unique role in ubiquitylation [54], DNA demethylation [35], and DNA repair [35], the colorectal cancer progression may involve the abnormal ubiquitylation and demethylation.

### 4.3. The Signal Propagation Path from the Early Stage Biomarker MAVS to the Late Stage Biomarker GFPT1

**Figure 4:**
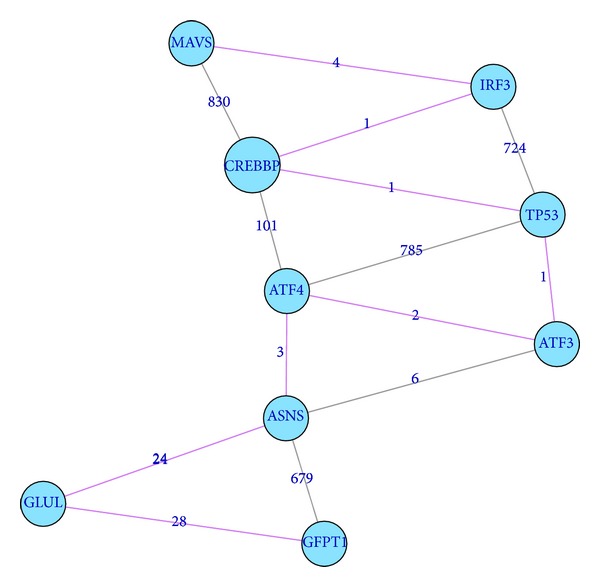
The signal propagation path from MAVS to GFPT1. The signal propagation path from MAVS to GFPT1 was MAVS → IRF3 → CREBBP → TP53 → ATF3 → ATF4 → ASNS → GLUL → GFPT1. The genes in the signal propagation path were mapped onto STRING network. The number on the edge was the edge weight. The edges on the signal propagation path were highlighted with pink color.

It is interesting to see that GFPT1 was ranked no. 1 among the late stage biomarkers although it was even not a biomarker in the early stage. We traced back in the signal propagation paths and found GFPT1 was the downstream of the following seven early stage biomarkers: MAVS, TET3, GAS1, ANGPTL4, MAP7D1, CEACAM1, and PGRMC1. Among the 158 early stage biomarkers, MAVS was ranked no. 4, but MAVS was not a late stage biomarker. The Pearson correlation test *P* value and Pearson correlation coefficient between the expression levels of MAVS and GFPT1 in the Dukes B patients and the Dukes C patients were 1.09*e* − 05 and 0.317, respectively. Shown in Figure 4 is the signal propagation path from MAVS to GFPT1 in the STRING network: MAVS → IRF3 → CREBBP → TP53 → ATF3 → ATF4 → ASNS → GLUL → GFPT1.

Mitochondrial antiviral signaling (MAVS) protein is important in innate immunity [13–15]. The antibody able to induce immune responses can be used to treat cancer [55]. Immune responses usually occur early in the cancer progression stage but later the cancer cells may develop an ability to escape the immune-mediated lysis [56]. This might explain why MAVS was an early stage biomarker, but not a late stage biomarker.

GFPT1 is the key enzyme in hexosamine synthesis pathway whose products have been implicated in O-linked N-acetylglucosamine (O-GlcNAc) protein modification, insulin resistance, and glucose toxicity [16, 17]. It is a molecular therapeutic target for type-2 diabetes [57, 58]. As a metabolic disease, cancer is always accompanied with impaired mitochondrial function and dysfunctional energy metabolism [59]. 

Accordingly, it is rational to deduce the signal propagation from MAVS to GFPT1 as follows: in mitochondria, as an important innate immunity protein, MAVS may response to colorectal cancer in a very early stage. Then as a signaling protein, it transmits its signal to GFPT1 that has close relationship with mitochondria. The perturbation of GFPT1 may cause the dysfunction of mitochondria in the energy metabolism. The fates of the cells may be doomed by the collapse of their energy systems. 

## 5. Conclusions

Our results indicated that the strong signals of early stage biomarkers would not necessarily disappear during the colorectal cancer progression, but might be transferred to other late stage biomarkers. This finding may provide useful insights for in-depth analyzing the signal propagation paths and helping to reveal the cellular mechanism of colorectal cancer aggravation. 

## Supplementary Material

Supplementary Material includes Table S1 - The 158 early stage biomarkers, Table S2 - The 284 late stage biomarkers, Table S3 - The 632 signal propagation paths from early stage biomarkers to late stage biomarkers and Table S4 - The 473 transition genes and the number of signal propagation paths crossed it.

## Figures and Tables

**Table 1:** The enriched GO terms of the 158 early stage biomarkers with adjusted *P* value less than 0.01.

Gene ontology	Number of input genes with the annotation	Adjusted *P* value
GO:0009607: response to biotic stimulus	16	0.001
GO:0006952: defense response	14	0.004
GO:0006955: immune response	13	0.004

**Table 2:** The enriched GO terms of the 284 late stage biomarkers with adjusted *P* value less than 0.01.

Gene ontology	Number of input genes with the annotation	Adjusted *P* value
GO:0006952: defense response	25	0.0002
GO:0006955: immune response	23	0.0002
GO:0016064: immunoglobulin mediated immune response	8	0.0006
GO:0006959: humoral immune response	9	0.001
GO:0009607: response to biotic stimulus	25	0.002
GO:0019730: antimicrobial humoral response	6	0.005

**Table 3:** The most enriched five GO terms of the 473 transition genes.

Gene ontology	Number of input genes with the annotation	Adjusted *P* value	Bayes factor
GO:0008283: cell proliferation	107	<0.0001	47
GO:0007154: cell communication	219	<0.0001	43
GO:0007165: signal transduction	191	<0.0001	43
GO:0051244: regulation of cellular physiological process	71	<0.0001	40
GO:0050794: regulation of cellular process	84	<0.0001	38
