# Supplementary material for: Signal Propagation in Protein Interaction Network during Colorectal Cancer Progression
Source: Biomed Res Int. 2013 Mar 20;2013:287019. doi: 10.1155/2013/287019 (PMC3615629; doi:10.1155/2013/287019)
Supplement: Supplementary file 1 — Supplementary Material includes Table S1 - The 158 early stage biomarkers, Table S2 - The 284 late stage biomarkers, Table S3 - The 632 signal propagation paths from early stage biomarkers to late stage biomarkers and Table S4 - The 473 transition genes and the number of signal propagation paths crossed it. [file 287019.f1.pdf]

Table S1 - The 158 early stage biomarkers

Order	Name	mRMR Score
1	CASP1	0.107
2	ZNF552	0.086
3	WDYHV1	0.082
4	MAVS	0.082
5	MBNL2	0.074
6	CHRNA3	0.069
7	PACS2	0.069
8	SLC11A1	0.065
9	SOX11	0.064
10	ZC3H12A	0.063
11	LGALS8	0.06
12	SYK	0.06
13	TTC7B	0.059
14	CHST2	0.057
15	FAM12B	0.055
16	MBTD1	0.056
17	MMP8	0.052
18	PRKCDBP	0.052
19	NTRK1	0.051
20	CDKN2A	0.05
21	PQBP1	0.049
22	SERPINB2	0.049
23	C18orf1	0.049
24	SLC17A7	0.047
25	LRP4	0.046
26	METTL8	0.045
27	RB1CC1	0.045
28	TET3	0.045
29	SALL2	0.045
30	DDX47	0.044
31	SLC22A5	0.044
32	FAM20B	0.045
33	CDON	0.044
34	SRBD1	0.045
35	CCL20	0.045
36	NKAIN2	0.043
37	LOC100130175	0.043
38	SAV1	0.043
39	ANKS4B	0.043

40	SART3	0.043
41	TSPYL4	0.042
42	C4orf29	0.043
43	PSMB9	0.043
44	CCDC83	0.042
45	CPA2	0.042
46	TMPRSS6	0.042
47	GPR177	0.042
48	UBE2CBP	0.041
49	SNAPC3	0.041
50	BAX	0.041
51	FCRL5	0.041
52	BEND6	0.04
53	PRF1	0.04
54	LEPRE1	0.041
55	SLC22A18	0.041
56	KRCC1	0.04
57	CCL7	0.04
58	GADD45GIP1	0.04
59	DIRAS3	0.04
60	TTC17	0.039
61	KDM4B	0.039
62	SCEL	0.038
63	LGI1	0.038
64	C4orf31	0.037
65	EBI3	0.037
66	TXNRD3	0.037
67	GAS1	0.037
68	GRIN3A	0.037
69	RFXAP	0.036
70	AKR1C3	0.036
71	RNF43	0.037
72	MRPL51	0.036
73	HIATL1	0.036
74	PIGR	0.036
75	DIS3L2	0.036
76	ZPBP	0.036
77	LOC90925	0.035
78	RPS2	0.036
79	LOC100129502	0.035
80	IL26	0.035

81	UAP1L1	0.034
82	HSPB1	0.035
83	IGKC	0.035
84	SAMD11	0.034
85	VIP	0.034
86	KIAA0562	0.034
87	MAGT1	0.034
88	UBXN7	0.033
89	PTGER1	0.033
90	CRB3	0.033
91	ATL3	0.033
92	IGLV4-60	0.033
93	OLIG1	0.033
94	C6orf211	0.033
95	COL8A1	0.033
96	MEG3	0.033
97	ANGPTL4	0.033
98	IGHD	0.032
99	ADAMTS16	0.032
100	OCA2	0.032
101	FASTKD1	0.032
102	CXCL14	0.032
103	SPATS2	0.032
104	TRAV8-3	0.032
105	BTBD3	0.032
106	JHDM1D	0.032
107	RABGEF1	0.032
108	PARVA	0.032
109	CCDC102B	0.032
110	IGJ	0.032
111	DYDC1	0.032
112	SLC2A3	0.032
113	GLTPD1	0.032
114	OXA1L	0.031
115	PRKAA2	0.031
116	C19orf61	0.031
117	TATDN2	0.031
118	KATNAL1	0.031
119	ZNF91	0.031
120	FAM53B	0.031
121	CYP4F2	0.031

122	ATP2B4	0.031
123	C6orf154	0.031
124	CD79A	0.031
125	EEF1DP3	0.031
126	COL4A5	0.03
127	PARG	0.03
128	RNF4	0.03
129	NS3BP	0.03
130	PLEKHB1	0.03
131	MAP7D1	0.03
132	COBLL1	0.03
133	GPR85	0.029
134	HLA-DQB2	0.029
135	PITX1	0.029
136	ALKBH6	0.029
137	C2orf61	0.029
138	SATB2	0.029
139	GMCL1	0.029
140	APOBEC1	0.029
141	NOP10	0.028
142	PLXNB3	0.028
143	PTGER3	0.028
144	WBSCR27	0.028
145	LOC339290	0.028
146	ZNF551	0.028
147	EEF1B2	0.028
148	LPXN	0.028
149	UBQLN2	0.028
150	ACSF2	0.028
151	CEACAM1	0.028
152	NFIB	0.028
153	PIKFYVE	0.028
154	SPATA12	0.028
155	DYNLRB2	0.028
156	PGRMC1	0.028
157	C3orf58	0.028
158	ZNF485	0.028

Table S2 - The 284 late stage biomarkers

Order	Name	mRMR Score
1	GFPT1	0.116
2	LOC402778	0.082
3	ANP32E	0.077
4	IKBKE	0.058
5	LOC400654	0.062
6	FCGR3B	0.06
7	FLJ14107	0.056
8	HOMEZ	0.056
9	FZD8	0.057
10	KCNQ2	0.05
11	PIK3AP1	0.048
12	TMCC3	0.046
13	STAG3	0.047
14	CRLS1	0.047
15	LYRM5	0.047
16	C14orf159	0.047
17	hCG_1820661	0.047
18	SNAPIN	0.046
19	CCDC68	0.047
20	SSR1	0.044
21	ALPK3	0.044
22	TMEM99	0.045
23	LRCH3	0.044
24	ZNF827	0.041
25	C9orf152	0.041
26	FBRS	0.04
27	ASF1A	0.04
28	VNN2	0.042
29	AQP10	0.04
30	GDPD3	0.041
31	MPPED1	0.039
32	MTERFD2	0.037
33	SAA3P	0.037
34	SOCS7	0.038
35	SMAD7	0.037
36	UQCC	0.037
37	ZZZ3	0.036
38	TAP2	0.036
39	LMTK3	0.035

40	ANKRD29	0.035
41	PPP1R3A	0.035
42	COPA	0.035
43	MTMR12	0.035
44	MID1	0.035
45	MARCKS	0.035
46	CABP5	0.034
47	CD55	0.034
48	ABHD15	0.034
49	KIAA0182	0.034
50	SPINK5	0.033
51	ITGA6	0.033
52	EPPK1	0.033
53	KIF1A	0.032
54	ZFHX3	0.033
55	PQLC1	0.032
56	CTSA	0.033
57	RNF138	0.032
58	GSDMD	0.031
59	FPR1	0.031
60	ATP6V1E2	0.031
61	CAMK2B	0.031
62	ZMYM4	0.031
63	GPR19	0.031
64	ZNF818P	0.031
65	TBCKL	0.03
66	TM2D2	0.03
67	SHANK2	0.03
68	RUNDC3A	0.03
69	CLEC2D	0.03
70	C1orf14	0.03
71	AMMECR1L	0.03
72	DEFA5	0.03
73	C17orf76	0.029
74	TMC2	0.029
75	SERPINB4	0.029
76	KLRD1	0.029
77	SLC38A9	0.029
78	SOS2	0.029
79	CXXC4	0.029
80	ADAM9	0.029

81	ARHGEF19	0.029
82	AVPI1	0.029
83	NSUN7	0.029
84	MBNL3	0.029
85	APBB1IP	0.028
86	MMGT1	0.028
87	HAVCR1	0.028
88	VPS4B	0.028
89	C8orf48	0.028
90	SLC16A6	0.028
91	TOX2	0.028
92	CYP2J2	0.028
93	ABCB1	0.028
94	DYNC1I2	0.028
95	SYTL3	0.027
96	CGGBP1	0.027
97	ORC3L	0.027
98	HMOX1	0.027
99	TFCP2	0.026
100	C20orf62	0.027
101	LOC730184	0.027
102	C9orf4	0.027
103	A1CF	0.027
104	IRF1	0.027
105	ARHGEF1	0.026
106	C20orf7	0.026
107	TREML1	0.026
108	TNFRSF18	0.026
109	MTMR3	0.026
110	FAIM3	0.026
111	NEB	0.026
112	TNFRSF1B	0.026
113	RNFT1	0.026
114	SUV420H1	0.026
115	AARSD1	0.026
116	RXRB	0.026
117	ASB6	0.025
118	PRO2012	0.025
119	SCML1	0.026
120	PREPL	0.026
121	RPRM	0.025

122	LOC100130460	0.025
123	PKP2	0.025
124	EDN3	0.025
125	ASS1	0.025
126	ITGAD	0.025
127	C6orf217	0.025
128	GFER	0.025
129	SLC39A14	0.025
130	TTLL7	0.024
131	UPP1	0.025
132	LYSMD2	0.024
133	CD177	0.024
134	TUBGCP4	0.024
135	RNF215	0.024
136	ACCN2	0.024
137	GRSF1	0.024
138	SLA	0.024
139	SOX8	0.024
140	CCDC127	0.024
141	ZCCHC2	0.024
142	KANK4	0.023
143	THADA	0.024
144	FREM1	0.024
145	C18orf10	0.023
146	ETV6	0.023
147	MYCN	0.023
148	PITPNA	0.023
149	RASGEF1B	0.023
150	SLCO1A2	0.023
151	TAX1BP3	0.023
152	CPA6	0.023
153	FAM181A	0.023
154	C8G	0.022
155	LOC100130522	0.022
156	PLUNC	0.022
157	RBM39	0.022
158	ZAP70	0.022
159	LOC339539	0.022
160	NAMPT	0.022
161	NGB	0.022
162	ATP6V1E1	0.022

163	LOC729991	0.022
164	RNF4	0.022
165	PPAPDC1B	0.022
166	LOC388210	0.022
167	CCL18	0.022
168	HRAS	0.022
169	MORC4	0.021
170	C9orf169	0.021
171	AADACL2	0.021
172	SCFD2	0.021
173	LOC283731	0.021
174	C14orf53	0.021
175	PKIB	0.021
176	THEMIS	0.021
177	SLC25A11	0.021
178	LTF	0.021
179	LOC153577	0.021
180	ATAD1	0.021
181	F2	0.021
182	HCN1	0.021
183	ST3GAL1	0.021
184	KIAA0947	0.021
185	LOC84740	0.021
186	BRAF	0.021
187	ENPP3	0.02
188	LOC100130275	0.02
189	ESRRG	0.02
190	SLC4A9	0.02
191	RASGRP2	0.02
192	NAT15	0.02
193	DNAJB8	0.02
194	LOC441242	0.02
195	PSMB3	0.02
196	SLC39A2	0.02
197	TRIM47	0.02
198	CRYZ	0.02
199	C15orf33	0.02
200	C2orf64	0.02
201	CD2	0.02
202	CEP57	0.02
203	POLR2C	0.02

204	MCM3APAS	0.02
205	MYEF2	0.02
206	TTC15	0.02
207	B3GNT7	0.02
208	LOC100130744	0.02
209	DACH2	0.02
210	SLC22A15	0.02
211	GALNT1	0.019
212	SEMA6C	0.02
213	BEX1	0.019
214	MTMR8	0.019
215	RBM41	0.019
216	GDNF	0.019
217	C1orf103	0.019
218	C1orf161	0.019
219	MMD	0.019
220	PURB	0.019
221	SEMA4B	0.019
222	ANKRD28	0.019
223	MYCL1	0.019
224	HLA-DRA	0.019
225	CCDC136	0.019
226	ZNF10	0.019
227	HPS5	0.019
228	ESX1	0.019
229	C7orf13	0.019
230	NECAB1	0.019
231	SEC31B	0.019
232	SP5	0.019
233	ZC3H15	0.019
234	CCDC153	0.019
235	LOC401913	0.019
236	SLC25A3	0.019
237	LPHN2	0.018
238	LMBR1L	0.018
239	C19orf45	0.018
240	HAS2	0.018
241	OGDHL	0.018
242	ZSWIM1	0.019
243	ZFP14	0.019
244	LOC284801	0.018

245	AMELY	0.018
246	SERPINA4	0.018
247	MED23	0.018
248	CXCL13	0.018
249	SFRS15	0.018
250	GATA3	0.018
251	MTERFD3	0.018
252	LOC283177	0.018
253	CAV3	0.018
254	GNA11	0.018
255	LOC338758	0.018
256	OCEL1	0.018
257	YLPM1	0.018
258	NCOA7	0.018
259	CLEC4E	0.018
260	RNF135	0.018
261	EIF1	0.018
262	FATE1	0.018
263	ZFYVE9	0.018
264	NUPR1	0.018
265	JAK2	0.018
266	IDH2	0.017
267	EGFL7	0.017
268	CRIM1	0.017
269	PTCH2	0.017
270	CASP9	0.017
271	CPSF7	0.017
272	NT5E	0.017
273	EGF	0.017
274	DRD1	0.017
275	DDX3Y	0.017
276	CD69	0.017
277	GCLC	0.017
278	UGDH	0.017
279	PIK3CG	0.017
280	SLITRK6	0.017
281	PTPRH	0.017
282	BCL11A	0.017
283	FMN2	0.017
284	CYTH2	0.017

Table S3 - The 632 signal propagation paths from early stage biomarkers to late stage biomarkers

Early stage biomarker	Late stage biomarker	Pearson correlation test p value	Pearson correlation test FDR	Pearson correlation coefficient	Shortest path	Path length (sum of edge weights)	Permutation FDR
SLC2A3	CD69	0	0	0.626900758	SLC2A3->SP1->EP300->STAT3->IL2->CD69	204	0
EBI3	ZAP70	1.28E-06	6.86E-05	0.347236703	EBI3->IL27RA->STAT1->JAK2->SHC1->ZAP70	7	0
CD79A	CD2	1.74E-09	2.91E-07	0.424410472	CD79A->SYK->LCK->CD2	6	0
CD79A	ZAP70	3.46E-10	7.52E-08	0.440624965	CD79A->SYK->CBL->ZAP70	5	0
RNF4	RNF4	0	0	1	RNF4->RNF4	0	0
PRF1	IRF1	6.68E-13	3.94E-10	0.496402019	PRF1->CD8A->IL2->STAT1->IRF1	9	0
PRF1	CD2	1.55E-15	2.70E-12	0.542713938	PRF1->CD8A->LCK->CD2	8	0
PSMB9	IRF1	2.22E-16	4.96E-13	0.557080516	PSMB9->PSMB7->UCHL5->PSMD4->UBC->EGFR->STAT1->IRF1	8	0
PSMB9	CD2	0	0	0.570225364	PSMB9->PSMB7->UCHL5->PSMD4->UBC->EGFR->GRB2->LCP2->LCK->CD2	10	0
PSMB9	ZAP70	1.19E-06	6.44E-05	0.34825263	PSMB9->PSMB7->UCHL5->PSMD4->UBC->EGFR->SHC1->ZAP70	7	0
SYK	SMAD7	3.38E-06	0.000149401	0.334015718	SYK->CBL->GRB2->ERBB2->CTNNB1->SMAD7	7	0
RNF43	BCL11A	0	0	0.585197535	RNF43->AKAP8L->DHX9->SF3A2->YBX1->TP53->SP1->ESR1->NCOA2->BCL11A	176	0
CASP1	IRF1	2.22E-16	4.96E-13	0.554916623	CASP1->IL18->IFNG->IFNGR1->STAT1->IRF1	6	0
CASP1	JAK2	7.44E-08	6.59E-06	0.382973243	CASP1->IL18->IFNG->IL2->STAT3->JAK2	6	0
CASP1	CD2	4.44E-15	6.04E-12	0.534985329	CASP1->IL18->IFNG->CD4->LCK->CD2	7	0
CASP1	ZAP70	1.77E-06	8.90E-05	0.342930182	CASP1->IL18->IFNG->CD4->ZAP70	7	0
CASP1	EGF	3.63E-06	0.000157382	0.332993074	CASP1->IL18->IFNG->IL2->STAT3->EGFR->EGF	7	0
SATB2	BCL11A	0	0	0.609237465	SATB2->HOXA2->CD1D->B2M->CD8A->IL2->STAT3->EP300->ESR1->NCOA2->BCL11A	141	0
CD79A	CXCL13	4.37E-13	2.74E-10	0.499846556	CD79A->SYK->LCK->CD4->CXCR4->CXCL13	16	5.00E-05
PRF1	TNFRSF1B	1.12E-13	8.37E-11	0.510627682	PRF1->CD8A->LCK->NFKBIA->CHUK->TRAF2->TNFRSF1B	12	5.00E-05
CD79A	CD69	1.10E-13	8.37E-11	0.510820388	CD79A->SYK->LCK->IL2->CD69	23	1.00E-04
PRF1	JAK2	5.10E-08	4.89E-06	0.387412908	PRF1->CD8A->IL2->STAT3->JAK2	8	1.00E-04
PRF1	ZAP70	1.83E-06	9.14E-05	0.342458478	PRF1->CD8A->LCK->ZAP70	8	1.00E-04
PSMB9	TNFRSF1B	1.61E-06	8.20E-05	0.344206323	PSMB9->PSMB7->UCHL5->PSMD4->UBC->RIPK1->TRAF2->TNFRSF1B	8	1.00E-04
EBI3	TNFRSF1B	1.52E-06	7.89E-05	0.344934855	EBI3->IL27RA->STAT1->EP300->RELA->CHUK->TRAF2->TNFRSF1B	9	0.00015
PRF1	KLRD1	8.00E-13	4.47E-10	0.494931163	PRF1->CD8A->LCK->SYK->TYROBP->KLRD1	37	0.00015
PARVA	MARCKS	1.50E-08	1.77E-06	0.401323009	PARVA->ILK->PXN->GIT1->ARHGEF7->CDC42->PLD1->PRKCA->MARCKS	10	0.00015
PSMB9	HLA-DRA	7.76E-13	4.41E-10	0.495176944	PSMB9->PSMB7->UCHL5->PSMD4->UBC->TP53->EP300->CREB1->CIITA->HLA-DRA	26	0.00015
EBI3	CD2	1.64E-05	0.000531378	0.310977657	EBI3->IL27RA->STAT1->JAK2->PTPN6->LCK->CD2	10	2.00E-04
PARVA	FZD8	9.84E-07	5.56E-05	0.350758246	PARVA->ILK->AKT1->GSK3B->LRP6->FZD8	10	2.00E-04
PSMB9	ITGA6	2.30E-09	3.67E-07	-0.421521154	PSMB9->PSMB7->UCHL5->PSMD4->UBC->EGFR->SRC->PTK2->PXN->ITGA4->ITGB1->ITGA6	11	2.00E-04
CD79A	PIK3CG	2.33E-08	2.53E-06	0.396401296	CD79A->SYK->VAV1->RAC1->AKT1->PIK3CG	11	0.00025
GAS1	A1CF	1.96E-13	1.36E-10	-0.506257527	GAS1->SHH->GLI2->BTRC->CTNNB1->PSEN1->APP->APOE->LDLR->APOB->APOBEC1->A1CF	46	0.00025
IGKC	CXCL13	4.88E-15	6.37E-12	0.534296591	IGKC->ERBB4->EGF->GRB2->LCP2->LCK->CD4->CXCR4->CXCL13	43	0.00025

IGKC	CD2	5.38E-12	2.21E-09	0.478890918	IGKC->ERBB4->EGF->GRB2->LCP2->LCK->CD2	33	0.00025
IGHD	CD69	4.44E-15	6.04E-12	0.534666347	IGHD->GHRH->GCG->LEP->STAT3->IL2->CD69	58	0.00025
IGHD	CXCL13	1.75E-14	1.79E-11	0.524769287	IGHD->GHRH->GCG->LEP->STAT3->IL2->IFNG->CD4->CXCR4->CXCL13	54	0.00025
IGHD	CD2	4.44E-16	8.68E-13	0.549783195	IGHD->GHRH->GCG->LEP->STAT3->IL2->LCK->CD2	45	0.00025
MAVS	HMOX1	6.75E-08	6.12E-06	-0.384127112	MAVS->IRF3->CREBBP->HIF1A->HMOX1	11	0.00025
CCL7	HMOX1	1.06E-08	1.32E-06	0.405126767	CCL7->CCR5->CD4->IFNG->IFNGR1->STAT1->EP300->HIF1A->HMOX1	13	0.00035
EBI3	HMOX1	2.55E-05	0.000750363	0.304270806	EBI3->IL27RA->STAT1->EP300->HIF1A->HMOX1	11	0.00035
RABGEF1	SOS2	7.78E-06	0.000289707	0.322112893	RABGEF1->RABEP1->RAB5A->EGFR->GRB2->SOS2	11	0.00035
SERPINB2	HMOX1	5.44E-08	5.17E-06	0.386653364	SERPINB2->PLAU->JUN->EP300->HIF1A->HMOX1	13	0.00035
PRF1	HMOX1	8.10E-08	7.01E-06	0.381961889	PRF1->CD8A->IL2->STAT3->EP300->HIF1A->HMOX1	14	0.00035
PRF1	CXCL13	2.23E-10	5.13E-08	0.444897138	PRF1->CD8A->CD4->CXCR4->CXCL13	17	0.00035
CASP1	CXCL13	5.01E-09	7.13E-07	0.413284923	CASP1->IL18->IFNG->CD4->CXCR4->CXCL13	15	0.00035
CEACAM1	GNA11	6.66E-11	1.93E-08	0.456335057	CEACAM1->PTPN11->GRB2->RET->GDNF->EDNRB->GNA11	36	4.00E-04
GAS1	LPHN2	1.29E-13	9.37E-11	0.509556943	GAS1->SHH->BMP4->BMPR1A->SMAD1->SMAD4->TGFBR1->FKBP1A->RYR1->HOMER1->SHANK2->LPHN2	86	4.00E-04
PRF1	HLA-DRA	1.40E-10	3.56E-08	0.449362216	PRF1->CD8A->IL2->STAT3->EP300->CREB1->CIITA->HLA-DRA	28	4.00E-04
PSMB9	CXCL13	1.52E-09	2.59E-07	0.425762805	PSMB9->PSMB7->UCHL5->PSMD4->UBC->EGFR->GRB2->LCP2->LCK->CD4->CXCR4->CXCL13	20	4.00E-04
EBI3	PIK3CG	2.14E-06	0.000102784	0.340293436	EBI3->IL27RA->STAT1->EP300->CREB1->AKT1->PIK3CG	14	0.00045
APOBEC1	A1CF	4.37E-07	2.86E-05	0.361276568	APOBEC1->A1CF	15	0.00045
PRF1	BEX1	6.52E-07	3.94E-05	0.356126438	PRF1->CD8A->IL2->SHC1->NGFR->BEX1	15	0.00045
ATP2B4	MARCKS	9.07E-10	1.67E-07	0.431045457	ATP2B4->CALM1->IQGAP1->CDC42->PLD1->PRKCA->MARCKS	24	0.00045
FCRL5	CXCL13	1.73E-14	1.79E-11	0.52490935	FCRL5->BCL9->CTNNB1->ERBB2->GRB2->LCP2->LCK->CD4->CXCR4->CXCL13	100	0.00045
IGJ	CD69	1.21E-12	6.26E-10	0.491541404	IGJ->IL2->CD69	74	0.00045
SLC11A1	HMOX1	1.36E-06	7.21E-05	0.34642529	SLC11A1->ZBTB17->MYC->EP300->HIF1A->HMOX1	15	5.00E-04
HSPB1	TFCP2	2.56E-05	0.000754145	-0.304177992	HSPB1->MAPKAPK2->MAPK14->CREB1->EP300->YY1->TFCP2	12	5.00E-04
PTGER1	NCOA7	3.10E-13	2.02E-10	-0.502611276	PTGER1->BDKRB2->KNG1->EGF->SRC->ESR1->NCOA7	103	5.00E-04
SLC22A18	PTPRH	9.68E-14	7.76E-11	0.511791911	SLC22A18->CDKN1C->MYOD1->SRF->PTPRH	108	5.00E-04
PSMB9	FCGR3B	1.21E-11	4.33E-09	0.471831178	PSMB9->PSMB7->UCHL5->PSMD4->UBC->EGFR->CBL->SYK->FCER1G->FCGR3B	56	5.00E-04
AKR1C3	A1CF	2.61E-12	1.22E-09	0.485060376	AKR1C3->PTGDS->PTGS2->IL6->STAT3->SRC->CTNNB1->PSEN1->APP->APOE->LDLR->APOB->APOBEC1->A1CF	71	5.00E-04
IGKC	CD69	3.33E-11	1.05E-08	0.462707233	IGKC->ERBB4->EGF->SRC->STAT3->IL2->CD69	47	5.00E-04
CASP1	HLA-DRA	9.05E-10	1.67E-07	0.431075723	CASP1->IL18->IFNG->IL2->STAT3->EP300->CREB1->CIITA->HLA-DRA	26	5.00E-04
IGJ	CXCL13	2.55E-12	1.21E-09	0.485272077	IGJ->IL2->CD8A->CD4->CXCR4->CXCL13	70	5.00E-04
ANGPTL4	CYP2J2	5.22E-13	3.18E-10	-0.498411954	ANGPTL4->LPL->APOE->APP->APBB1->KAT5->PLA2G4A->CYP2J2	103	0.00055
ATP2B4	LPHN2	1.22E-12	6.26E-10	0.491446424	ATP2B4->CALM1->IQGAP1->RAC1->AKT1->MTOR->FKBP1A->RYR1->HOMER1->SHANK2->LPHN2	93	0.00055
CASP1	PIK3CG	6.19E-06	0.000243487	0.325421407	CASP1->IL18->IFNG->IL2->STAT3->RAC1->AKT1->PIK3CG	14	0.00055
EBI3	HLA-DRA	4.63E-09	6.67E-07	0.414140884	EBI3->IL27RA->STAT1->EP300->CREB1->CIITA->HLA-DRA	25	6.00E-04
GAS1	KLRD1	1.27E-10	3.31E-08	0.450263555	GAS1->SHH->FGF8->FGFR1->PLCG1->CBL->SYK->TYROBP->KLRD1	42	6.00E-04
PRF1	FCGR3B	7.83E-11	2.19E-08	0.454834104	PRF1->CD8A->LCK->SYK->FCER1G->FCGR3B	57	6.00E-04

CASP1	CD69	6.10E-08	5.70E-06	0.385308469	CASP1->IL18->IFNG->IL2->CD69	21	6.00E-04
SATB2	RBM39	1.44E-12	7.15E-10	0.490071558	SATB2->HOXA2->CD1D->B2M->CD8A->LCK->CD2->CD2BP2->SF3A2->RBM39	110	6.00E-04
CEACAM1	A1CF	1.71E-10	4.18E-08	0.447449775	CEACAM1->PTPN11->GRB2->ERBB2->CTNNB1->PSEN1->APP->APOE->LDLR->APOB->APOBEC1->A1CF	47	0.00065
CEACAM1	EDN3	7.12E-08	6.40E-06	0.383483001	CEACAM1->PTPN11->GRB2->RET->GDNF->EDNRB->EDN3	22	0.00065
PTGER1	KCNQ2	4.81E-12	2.01E-09	0.47984446	PTGER1->BDKRB2->KNG1->EGF->SRC->ESR1->EP300->CREB1->CAMK2B->CALM1->KCNQ2	100	0.00065
SERPINB2	BCL11A	6.83E-12	2.70E-09	-0.476817364	SERPINB2->PLAU->JUN->EP300->ESR1->NCOA2->BCL11A	73	0.00065
MAVS	SHANK2	2.13E-08	2.36E-06	0.397403786	MAVS->IRF3->CREBBP->CREB1->AKT1->MTOR->FKBP1A->RYR1->HOMER1->SHANK2	25	0.00065
CASP1	ASS1	3.31E-08	3.38E-06	0.392393444	CASP1->IL18->IFNG->IL2->STAT3->RAC1->AKT1->NOS3->ARG2->OTC->ASS1	24	0.00065
CD79A	HLA-DRA	1.17E-08	1.42E-06	0.404109604	CD79A->SYK->VAV1->RAC1->AKT1->CREB1->CIITA->HLA-DRA	26	7.00E-04
APOBEC1	TFCP2	8.41E-09	1.08E-06	0.407701882	APOBEC1->APOB->LDLR->APOE->APP->PSEN1->CTNNB1->GSK3B->TP53->YY1->TFCP2	28	7.00E-04
PSMB9	CD69	6.75E-08	6.12E-06	0.384113473	PSMB9->PSMB7->UCHL5->PSMD4->UBC->EGFR->STAT3->IL2->CD69	24	7.00E-04
RNF43	ABCB1	4.44E-16	8.68E-13	0.552683482	RNF43->AKAP8L->DHX9->SF3A2->YBX1->TP53->ABCB1	150	7.00E-04
CEACAM1	CYP2J2	3.70E-11	1.15E-08	0.461755841	CEACAM1->PTPN11->EGFR->UBC->TP53->KAT5->PLA2G4A->CYP2J2	75	0.00075
CCL7	FCGR3B	1.93E-10	4.56E-08	0.446269883	CCL7->CCR5->CD4->LCK->SYK->FCER1G->FCGR3B	55	0.00075
GAS1	HMOX1	4.55E-07	2.97E-05	0.360744181	GAS1->SHH->FGF8->FGFR1->PLCG1->EGFR->UBC->HIF1A->HMOX1	19	0.00075
GAS1	KANK4	3.07E-12	1.37E-09	0.483685968	GAS1->SHH->FGF8->FGFR1->PLCG1->EGFR->UBC->TP53->TBP->POLR2A->POLR2H->POLR3D->POLR3F->POLR3C->KANK4	136	0.00075
MAVS	CRLS1	2.09E-13	1.41E-10	0.505753936	MAVS->IRF3->CREBBP->TP53->BCL2->BNIP2->CDC42->PLD1->CDS2->CRLS1	156	0.00075
SATB2	ENPP3	9.77E-15	1.13E-11	0.529167499	SATB2->HOXA2->CD1D->B2M->CD8A->IL2->IL5->IL3->ENPP3	172	0.00075
SLC2A3	FCGR3B	2.44E-15	4.02E-12	0.539204886	SLC2A3->SP1->ESR1->SRC->CBL->SYK->FCER1G->FCGR3B	238	8.00E-04
SLC2A3	FPR1	2.22E-16	4.96E-13	0.557121839	SLC2A3->SP1->TP53->NPM1->NCL->S100A11->ANXA1->FPR1	222	8.00E-04
SLC2A3	KLRD1	6.20E-14	5.10E-11	0.515223784	SLC2A3->SP1->ESR1->SRC->CBL->SYK->TYROBP->KLRD1	218	8.00E-04
SLC2A3	HLA-DRA	2.89E-15	4.51E-12	0.537670168	SLC2A3->SP1->EP300->CREB1->CIITA->HLA-DRA	205	8.00E-04
SLC11A1	FCGR3B	8.34E-11	2.31E-08	0.454237845	SLC11A1->ZBTB17->MYC->EP300->STAT3->EGFR->CBL->SYK->FCER1G->FCGR3B	62	8.00E-04
RNF43	SCML1	6.06E-14	5.10E-11	0.515391773	RNF43->AKAP8L->DHX9->SF3A2->YBX1->TP53->HDAC1->SIN3A->MECP2->CDKL5->NHS->SCML1	234	8.00E-04
NOP10	TFCP2	1.50E-05	0.000495833	-0.312373076	NOP10->DKC1->RUVBL1->KAT5->TP53->YY1->TFCP2	16	0.00085
IGHD	PIK3CG	3.56E-10	7.68E-08	0.440345527	IGHD->GHRH->POMC->NR4A1->AKT1->PIK3CG	49	0.00085
CEACAM1	CLEC4E	5.95E-14	5.10E-11	-0.515541273	CEACAM1->PTPN11->EGFR->UBC->IKBKG->BCL10->CARD9->CLEC4E	275	9.00E-04
SLC11A1	PIK3AP1	1.82E-10	4.37E-08	0.446858197	SLC11A1->ZBTB17->MYC->MAPK1->GRB2->PIK3R1->PIK3AP1	66	9.00E-04
CHST2	CD69	1.33E-15	2.45E-12	0.543287197	CHST2->SELL->CD34->MYB->CREBBP->TP53->EP300->STAT3->IL2->CD69	272	9.00E-04
CHST2	HLA-DRA	7.99E-15	9.62E-12	0.530515724	CHST2->SELL->CD34->MYB->CREBBP->CREB1->CIITA->HLA-DRA	271	9.00E-04
NOP10	A1CF	1.22E-08	1.47E-06	-0.403588966	NOP10->DKC1->RUVBL1->CTNNB1->PSEN1->APP->APOE->LDLR->APOB->APOBEC1->A1CF	40	9.00E-04
NOP10	TNFRSF18	2.68E-06	0.000123487	0.337245108	NOP10->DKC1->TERT->AKT1->RAC1->STAT3->IL2->TNFRSF18	18	9.00E-04
NOP10	KLRD1	1.26E-08	1.50E-06	0.40328604	NOP10->DKC1->TERT->AKT1->RAC1->VAV1->SYK->TYROBP->KLRD1	39	9.00E-04
IGHD	HLA-DRA	1.94E-10	4.56E-08	0.446233583	IGHD->GHRH->GCG->LEP->STAT3->EP300->CREB1->CIITA->HLA-DRA	61	9.00E-04

CCL7	CD69	1.08E-06	5.95E-05	0.349554402	CCL7->CCR5->CD4->CD8A->IL2->CD69	22	0.00095
ANGPTL4	HMOX1	1.00E-09	1.82E-07	0.430022593	ANGPTL4->LPL->APOE->APP->PSEN1->CTNNB1->GSK3B->TP53->HIF1A->HMOX1	49	0.00095
PRF1	CD69	1.38E-06	7.27E-05	0.346294404	PRF1->CD8A->IL2->CD69	23	0.00095
PRF1	SLA	1.76E-11	6.05E-09	0.468446673	PRF1->CD8A->IL2->STAT3->EP300->MYOD1->TCF3->TAL1->LMO2->LDB1->LHX3->IFT172->SLA	127	0.00095
NOP10	UPP1	5.71E-10	1.14E-07	0.435678467	NOP10->DKC1->RUVBL1->KAT5->TP53->RRM2B->RRM1->RRM2->TYMS->TK1->CDA->TYMP->UPP1	51	0.00095
GAS1	CCL18	1.07E-10	2.83E-08	0.451902229	GAS1->SHH->FGF8->FGFR1->FRS2->GRB2->LCP2->LCK->CD4->CCR5->CCL13->CCR3->CCL18	81	0.001
GAS1	SLA	3.48E-11	1.09E-08	0.462315159	GAS1->SHH->GLI2->IFT172->SLA	129	0.001
SERPINB2	A1CF	4.39E-09	6.45E-07	-0.414694299	SERPINB2->PLAU->NFKB1->NFKBIA->BTRC->CTNNB1->PSEN1->APP->APOE->LDLR->APOB->APOBEC1->A1CF	42	0.001
PARVA	LPHN2	1.22E-10	3.20E-08	0.450682714	PARVA->ILK->AKT1->MTOR->FKBP1A->RYR1->HOMER1->SHANK2->LPHN2	76	0.001
MAVS	A1CF	1.17E-08	1.42E-06	0.404054378	MAVS->IRF3->CREBBP->CTNNB1->PSEN1->APP->APOE->LDLR->APOB->APOBEC1->A1CF	41	0.001
SATB2	CD55	3.90E-11	1.20E-08	-0.461266693	SATB2->HOXA2->CD1D->B2M->CD8A->LCK->SYK->CD19->CR2->C3->CD55	120	0.001
SLC2A3	CCL18	5.29E-13	3.18E-10	0.498301001	SLC2A3->SP1->EP300->RELA->NFKB1->CCL5->CCR3->CCL18	256	0.00105
CEACAM1	HMOX1	3.52E-06	0.000153605	-0.333450511	CEACAM1->PTPN11->EGFR->UBC->HIF1A->HMOX1	19	0.00105
SLC22A18	GNA11	1.86E-10	4.45E-08	0.446614204	SLC22A18->CDKN1C->MYOD1->EP300->JUN->EDN1->EDNRB->GNA11	81	0.00105
CRB3	MARCKS	5.51E-07	3.46E-05	-0.358311949	CRB3->PARD6A->CDC42->PLD1->PRKCA->MARCKS	26	0.00105
PSMB9	KLRD1	7.99E-08	6.96E-06	0.382130458	PSMB9->PSMB7->UCHL5->PSMD4->UBC->EGFR->CBL->SYK->TYROBP->KLRD1	36	0.00105
RNF43	CXCL13	4.84E-11	1.46E-08	-0.459284305	RNF43->AKAP8L->DHX9->SF3A2->CD2BP2->CD2->LCK->CD4->CXCR4->CXCL13	119	0.00105
CASP1	ZFHX3	2.27E-09	3.63E-07	-0.421664979	CASP1->IL18->IL1B->IL8->RELA->CREBBP->MYB->ZFHX3	48	0.00105
SLC2A3	APBB1IP	3.31E-12	1.46E-09	0.48305194	SLC2A3->SP1->ESR1->SRC->PTK2->TLN1->APBB1IP	234	0.0011
PGRMC1	SCML1	2.83E-12	1.28E-09	0.484389946	PGRMC1->M6PR->IGF1->IGF1R->ESR1->SP1->HDAC1->SIN3A->MECP2->CDKL5->NHS->SCML1	208	0.0011
PIGR	CXCL13	4.18E-06	0.000176829	0.331031366	PIGR->CD79A->SYK->LCK->CD4->CXCR4->CXCL13	20	0.0011
SATB2	MID1	6.95E-12	2.72E-09	0.476658864	SATB2->HOXA2->CD1D->B2M->CD8A->IL2->STAT3->EP300->SP1->SMAD3->RUNX2->SP7->MID1	146	0.0011
CCL7	CCL18	1.11E-09	1.96E-07	0.429045553	CCL7->CCR3->CCL18	67	0.00115
EBI3	SNAPIN	4.17E-07	2.75E-05	0.361854206	EBI3->IL27RA->STAT1->JAK2->GRB2->PDGFRB->SLC9A3R1->CFTR->STX1A->SNAP25->SNAPIN	29	0.00115
ANGPTL4	FPR1	2.80E-10	6.22E-08	0.442678475	ANGPTL4->LPL->APOE->APP->PSEN1->CTNNB1->GSK3B->TP53->NPM1->NCL->S100A11->ANXA1->FPR1	80	0.00115
CHRNA3	MARCKS	2.56E-08	2.72E-06	0.395326163	CHRNA3->PSMA4->UCHL5->PSMD4->UBC->EGFR->CBL->ARHGEF7->CDC42->PLD1->PRKCA->MARCKS	46	0.00115
PRF1	PIK3AP1	9.26E-10	1.69E-07	0.430843795	PRF1->CD8A->LCK->LCP2->GRB2->PIK3R1->PIK3AP1	63	0.00115
NOP10	RBM39	2.11E-08	2.34E-06	-0.397538201	NOP10->DKC1->RUVBL1->KAT5->TP53->YBX1->SF3A2->RBM39	47	0.00115
PTGER3	FZD8	2.93E-09	4.52E-07	0.418971416	PTGER3->KNG1->EGF->ERBB2->CTNNB1->AXIN1->LRP6->FZD8	58	0.00115
RNF43	A1CF	1.01E-11	3.75E-09	0.47342889	RNF43->AKAP8L->DHX9->SF3A2->CD2BP2->CD2->LCK->LCP2->GRB2->ERBB2->CTNNB1->PSEN1->APP->APOE->LDLR->APOB->APOBEC1->A1CF	144	0.00115
MAVS	GNA11	4.24E-07	2.78E-05	0.361661435	MAVS->IRF3->CREBBP->CREB1->FOS->EDN1->EDNRB->GNA11	29	0.00115
IGJ	CD2	1.35E-09	2.32E-07	0.427009846	IGJ->IL2->LCK->CD2	61	0.00115
PTGER3	LPHN2	6.98E-11	2.00E-08	0.45589728	PTGER3->KNG1->KLKB1->A2M->TGFB1->TGFBR1->FKBP1A->RYR1->HOMER1->SHANK2->LPHN2	128	0.0012
SYK	ESRRG	8.08E-07	4.72E-05	0.353344265	SYK->CBL->EGFR->UBC->TP53->SIRT1->PPARGC1A->ESRRG	29	0.0012
MAP7D1	SLA	5.59E-12	2.27E-09	0.478558607	MAP7D1->ARTN->GFRA1->RET->SHC1->IGF1R->ESR1-	229	0.00125

					>BRCA1->RBBP8->LMO4->LDB1->LHX3->IFT172->SLA		
ANGPTL4	SNAPIN	2.13E-09	3.43E-07	0.422310071	ANGPTL4->LPL->APOE->APP->PSEN1->CTNNB1->ERBB2->GRB2->PDGFRB->SLC9A3R1->CFTR->STX1A->SNAP25->SNAPIN	66	0.0013
SAV1	A1CF	2.07E-10	4.79E-08	-0.445614751	SAV1->RASSF1->HRAS->SOS1->GRB2->ERBB2->CTNNB1->PSEN1->APP->APOE->LDLR->APOB->APOBEC1->A1CF	95	0.0013
NOP10	CXCL13	4.41E-06	0.000185849	0.330264623	NOP10->DKC1->TERT->AKT1->RAC1->VAV1->LCP2->LCK->CD4->CXCR4->CXCL13	23	0.0013
ATP2B4	HRAS	4.63E-06	0.000192648	-0.329565653	ATP2B4->CALM1->IQGAP1->RAC1->AKT1->PIK3CA->HRAS	23	0.0013
ATP2B4	SMAD7	5.69E-06	0.000227805	0.326633752	ATP2B4->CALM1->CAMK2B->CREB1->EP300->SMAD2->TGFBR1->SMAD7	23	0.0013
RNF43	UPP1	3.13E-11	1.01E-08	-0.463292255	RNF43->AKAP8L->DHX9->SF3A2->YBX1->TP53->RRM2B->RRM1->RRM2->TYMS->TK1->CDA->TYMP->UPP1	152	0.0013
RB1CC1	RBM39	2.37E-09	3.76E-07	0.421196345	RB1CC1->PTK2->GRB2->LCP2->LCK->CD2->CD2BP2->SF3A2->RBM39	65	0.00135
GAS1	CD69	1.45E-06	7.59E-05	0.345563219	GAS1->SHH->FGF8->FGFR1->PLCG1->EGFR->STAT3->IL2->CD69	31	0.00135
NOP10	SHANK2	4.69E-06	0.000194667	-0.329379909	NOP10->DKC1->TERT->AKT1->MTOR->FKBP1A->RYR1->HOMER1->SHANK2	24	0.00135
CEACAM1	FCGR3B	5.48E-09	7.59E-07	-0.412330378	CEACAM1->PTPN11->GRB2->BLNK->SYK->FCER1G->FCGR3B	63	0.0014
SLC11A1	FPR1	7.79E-08	6.82E-06	0.382421258	SLC11A1->ZBTB17->MYC->EP300->TP53->NPM1->NCL->S100A11->ANXA1->FPR1	47	0.0014
SAV1	JAK2	4.78E-09	6.86E-07	0.413792328	SAV1->RASSF1->HRAS->SOS1->PTPN11->JAK2	61	0.0014
PSMB9	ZFHX3	7.79E-08	6.82E-06	-0.382427639	PSMB9->PSMB7->UCHL5->PSMD4->UBC->TP53->CREBBP->MYB->ZFHX3	47	0.0014
MAVS	FCGR3B	8.55E-09	1.10E-06	-0.407523127	MAVS->IRF3->CREBBP->HIF1A->UBC->EGFR->CBL->SYK->FCER1G->FCGR3B	59	0.0014
MAVS	FPR1	1.42E-07	1.11E-05	-0.37523462	MAVS->IRF3->CREBBP->TP53->NPM1->NCL->S100A11->ANXA1->FPR1	43	0.0014
PTGER1	CRIM1	5.45E-11	1.61E-08	-0.458196402	PTGER1->BDKRB2->KNG1->EGF->SRC->ESR1->EP300->SMAD2->SMAD4->SMAD1->BMPR1A->BMP4->CRIM1	163	0.00145
PRF1	CCL18	2.68E-09	4.20E-07	0.419904779	PRF1->CD8A->CD4->CCR5->CCL5->CCR3->CCL18	73	0.00145
CXCL14	DYNC1I2	1.33E-11	4.67E-09	0.470969799	CXCL14->CXCR4->CD4->IFNG->IL2->STAT3->EP300->TP53->CHEK1->CLSPN->PLK1->NUDC->PAFAH1B1->DCTN1->DCTN2->DYNC1I2	210	0.00145
SYK	A1CF	8.91E-07	5.11E-05	0.352061463	SYK->CBL->GRB2->ERBB2->CTNNB1->PSEN1->APP->APOE->LDLR->APOB->APOBEC1->A1CF	37	0.00145
RNF43	PIK3AP1	5.03E-11	1.50E-08	-0.458935032	RNF43->AKAP8L->DHX9->SF3A2->CD2BP2->CD2->LCK->LCP2->GRB2->PIK3R1->PIK3AP1	164	0.00145
SATB2	A1CF	1.67E-10	4.12E-08	0.447647641	SATB2->HOXA2->CD1D->B2M->CD8A->IL2->STAT3->SRC->CTNNB1->PSEN1->APP->APOE->LDLR->APOB->APOBEC1->A1CF	110	0.00145
CEACAM1	KLRD1	2.79E-07	1.98E-05	-0.36693184	CEACAM1->PTPN11->GRB2->BLNK->SYK->TYROBP->KLRD1	43	0.0015
EBI3	RASGRP2	7.66E-09	1.01E-06	0.408712815	EBI3->IL27RA->STAT1->EP300->HIF1A->HSP90AA1->RAF1->KRAS->RASGRP2	68	0.0015
ANGPTL4	FCGR3B	6.29E-10	1.21E-07	0.434716752	ANGPTL4->LPL->APOE->APP->PSEN1->CTNNB1->ERBB2->GRB2->VAV1->SYK->FCER1G->FCGR3B	94	0.0015
NOP10	CD69	3.43E-06	0.000151157	0.333793133	NOP10->DKC1->TERT->AKT1->RAC1->STAT3->IL2->CD69	28	0.0015
RFXAP	TAX1BP3	1.63E-07	1.23E-05	-0.373578738	RFXAP->RFXANK->CIITA->CREB1->AKT1->RAC1->ARHGDIA->RHOA->RTKN->TAX1BP3	44	0.0015
GAS1	SHANK2	2.64E-06	0.00012232	-0.337455104	GAS1->SHH->BMP4->BMPR1A->SMAD1->SMAD4->TGFBR1->FKBP1A->RYR1->HOMER1->SHANK2	30	0.00155
PRF1	SHANK2	8.46E-06	0.000310627	-0.320876781	PRF1->CD8A->IL2->STAT3->EP300->SMAD2->TGFBR1->FKBP1A->RYR1->HOMER1->SHANK2	26	0.00155
SLC2A3	NAMPT	3.28E-11	1.05E-08	0.462864705	SLC2A3->SP1->EP300->STAT3->IL6->NAMPT	223	0.0016
SLC11A1	ARHGEF19	8.34E-07	4.84E-05	-0.352930061	SLC11A1->ZBTB17->MYC->CTNNB1->DVL1->DAAM1->ARHGEF19	41	0.0016
ANGPTL4	A1CF	2.19E-08	2.40E-06	-0.397076744	ANGPTL4->LPL->APOB->APOBEC1->A1CF	65	0.0016

CHST2	HMOX1	2.05E-11	6.88E-09	0.467114629	CHST2->SELL->CD34->MYB->CREBBP->HIF1A->HMOX1	257	0.0016
FCRL5	CD2	7.81E-10	1.47E-07	0.432559934	FCRL5->BCL9->CTNNB1->ERBB2->GRB2->LCP2->LCK->CD2	90	0.0016
CASP1	NAMPT	5.44E-07	3.42E-05	0.358474195	CASP1->IL18->IL1B->IL6->NAMPT	42	0.0016
CEACAM1	PTPRH	4.12E-08	4.08E-06	0.389871708	CEACAM1->PTPN11->MAPK3->ELK1->SRF->PTPRH	64	0.00165
CD79A	SNAPIN	1.26E-05	0.000426426	0.315016403	CD79A->BLNK->GRB2->PDGFRB->SLC9A3R1->CFTR->STX1A->SNAP25->SNAPIN	26	0.00165
LEPRE1	LPHN2	1.30E-09	2.24E-07	0.427430999	LEPRE1->PPIB->BSG->MMP1->TIMP1->TGFB1->TGFBR1->FKBP1A->RYR1->HOMER1->SHANK2->LPHN2	96	0.00165
GAS1	TAX1BP3	7.21E-07	4.30E-05	0.354818627	GAS1->SHH->FGF8->FGFR1->FRS2->GRB2->VAV1->RAC1->ARHGDIA->RHOA->RTKN->TAX1BP3	43	0.00165
FCRL5	CD69	5.88E-10	1.16E-07	0.435388774	FCRL5->BCL9->CTNNB1->CCND1->STAT3->IL2->CD69	105	0.00165
IGKC	ZAP70	7.56E-06	0.000285698	0.322517626	IGKC->ERBB4->EGF->EGFR->SHC1->ZAP70	30	0.00165
RNF43	VNN2	2.30E-11	7.57E-09	-0.466073286	RNF43->AKAP8L->DHX9->SF3A2->CD2BP2->CD2->LCK->NFKBIA->NFKB1->ICAM1->ITGB2->VNN2	271	0.00165
CEACAM1	SHANK2	4.58E-06	0.000190817	0.329720073	CEACAM1->PTPN11->JAK2->STAT3->EP300->SMAD2->TGFBR1->FKBP1A->RYR1->HOMER1->SHANK2	32	0.0017
EBI3	KCNQ2	1.57E-06	8.07E-05	0.344487725	EBI3->IL27RA->STAT1->EP300->CREB1->CAMK2B->CALM1->KCNQ2	40	0.0017
RNF43	JAK2	5.14E-10	1.05E-07	-0.436723353	RNF43->AKAP8L->DHX9->SF3A2->CD2BP2->CD2->LCK->PTPN6->JAK2	110	0.0017
CCL7	HLA-DRA	1.15E-05	0.000395266	0.316383976	CCL7->CCR5->CD4->IFNG->IFNGR1->STAT1->EP300->CREB1->CIITA->HLA-DRA	27	0.00175
CEACAM1	CCL18	3.78E-09	5.73E-07	-0.416306236	CEACAM1->PTPN11->GRB2->LCP2->LCK->CD4->CCR5->CCL13->CCR3->CCL18	82	0.0018
CD79A	SLA	6.60E-10	1.26E-07	0.434235739	CD79A->BLNK->GRB2->ERBB2->CTNNB1->TCF4->TAL1->LMO2->LDB1->LHX3->IFT172->SLA	124	0.0018
SERPINB2	ABCB1	2.09E-07	1.53E-05	-0.370475021	SERPINB2->PLAU->JUN->MAPK8->TP53->ABCB1	48	0.0018
IGHD	KLRD1	2.94E-08	3.06E-06	0.393740885	IGHD->GHRH->GCG->LEP->STAT3->EGFR->CBL->SYK->TYROBP->KLRD1	72	0.0018
GAS1	HLA-DRA	6.78E-06	0.000261642	0.324095318	GAS1->SHH->FGF8->FGFR1->FRS2->PTPN11->STAT1->EP300->CREB1->CIITA->HLA-DRA	34	0.00185
SERPINB2	RBM39	2.78E-07	1.98E-05	-0.366963937	SERPINB2->PLAU->JUN->MAPK8->TP53->YBX1->SF3A2->RBM39	46	0.00185
NFIB	CRIM1	1.56E-10	3.88E-08	0.448308638	NFIB->HNF1A->HNF4A->SMAD4->SMAD1->BMPR1A->BMP4->CRIM1	146	0.00185
IGHD	ZAP70	1.23E-06	6.63E-05	0.347791003	IGHD->GHRH->GCG->LEP->STAT3->JAK2->SHC1->ZAP70	43	0.00185
RB1CC1	SLC25A11	5.46E-10	1.09E-07	-0.436130536	RB1CC1->PTK2->PTEN->TP53->BCL2->BNIP3->BCKDHB->DLD->OGDH->SLC25A11	134	0.0019
SAV1	CD55	1.42E-09	2.43E-07	0.426475735	SAV1->RASSF1->HRAS->SOS1->GRB2->VAV1->CD19->CR2->C3->CD55	106	0.00195
PTGER1	ARHGEF19	3.82E-09	5.78E-07	-0.416171277	PTGER1->BDKRB2->KNG1->EGF->ERBB2->CTNNB1->DVL1->DAAM1->ARHGEF19	95	0.002
MAP7D1	ST3GAL1	7.25E-11	2.06E-08	0.455550461	MAP7D1->ARTN->GFRA1->RET->GRB2->LCP2->LCK->CD2->CD2BP2->SF3A2->SNRPE->LSM4->EXOSC10->CHPF->B4GALNT1->ST8SIA1->ST3GAL1	250	0.002
IGKC	PIK3CG	9.12E-06	0.000327379	0.319785463	IGKC->ERBB4->EGF->EGFR->SOS1->HRAS->PIK3CG	37	0.00215
CCL7	ARHGEF19	8.79E-06	0.000319499	-0.320318201	CCL7->CCR5->CD4->LCK->LCP2->GRB2->ERBB2->CTNNB1->DVL1->DAAM1->ARHGEF19	39	0.0022
CCL7	A1CF	7.26E-06	0.000276534	-0.32311507	CCL7->CCR5->CD4->LCK->LCP2->GRB2->ERBB2->CTNNB1->PSEN1->APP->APOE->LDLR->APOB->APOBEC1->A1CF	41	0.0022
NOP10	ABCB1	6.02E-07	3.70E-05	-0.357159241	NOP10->DKC1->RUVBL1->KAT5->TP53->ABCB1	49	0.0022
MAP7D1	LPHN2	1.05E-10	2.81E-08	0.452064977	MAP7D1->ARTN->GFRA1->RET->GRB2->VAV1->RAC1->AKT1->MTOR->FKBP1A->RYR1->HOMER1->SHANK2->LPHN2	184	0.0022
SATB2	CTSA	4.22E-09	6.22E-07	0.415136146	SATB2->HOXA2->CD1D->CTSA	104	0.0022
SATB2	ABCB1	1.85E-09	3.06E-07	0.423765915	SATB2->HOXA2->CD1D->B2M->CD8A->IL2->STAT3->EP300->TP53->ABCB1	117	0.0022
LEPRE1	PKP2	2.52E-06	0.00011848	-0.338104154	LEPRE1->PPIB->BSG->MMP1->JUN->MAPK8->TP53-	44	0.00225

					>GSK3B->CTNNB1->CDH2->JUP->DSP->PKP2		
SAV1	ENPP3	2.89E-10	6.38E-08	-0.442365609	SAV1->RASSF1->HRAS->SOS1->PTPN11->JAK2->CSF2RB->IL3->ENPP3	160	0.00225
RNF43	NT5E	5.38E-10	1.09E-07	-0.43627473	RNF43->AKAP8L->DHX9->SF3A2->YBX1->TP53->USP7->GMPS->IMPDH2->NT5E	154	0.00225
SLC11A1	KLRD1	6.82E-06	0.000262725	0.3240177	SLC11A1->ZBTB17->MYC->EP300->STAT3->EGFR->CBL->SYK->TYROBP->KLRD1	42	0.0023
PRF1	CLEC4E	8.79E-11	2.39E-08	0.45374436	PRF1->CD8A->LCK->NFKBIA->IKBKG->BCL10->CARD9->CLEC4E	269	0.0023
IGHD	THEMIS	1.05E-10	2.81E-08	0.452093266	IGHD->GHRH->GCG->LEP->STAT3->IL2->THEMIS	225	0.0023
PRKAA2	MYEF2	1.50E-06	7.76E-05	0.34516758	PRKAA2->MTOR->AKT1->MDM2->RB1->RBBP4->SIN3A->HDAC9->MYEF2	46	0.0023
PSMB9	THEMIS	1.75E-10	4.26E-08	0.447195217	PSMB9->PSMB7->UCHL5->PSMD4->UBC->EGFR->STAT3->IL2->THEMIS	191	0.00235
CASP1	THEMIS	1.54E-10	3.86E-08	0.448427637	CASP1->IL18->IFNG->IL2->THEMIS	188	0.00235
IGJ	PIK3CG	1.59E-07	1.21E-05	0.373867805	IGJ->IL2->STAT3->RAC1->AKT1->PIK3CG	67	0.00235
SLC11A1	ABCB1	8.83E-07	5.08E-05	-0.352176089	SLC11A1->ZBTB17->MYC->EP300->TP53->ABCB1	51	0.0024
PSMB9	KCNQ2	9.45E-06	0.000336822	0.319258234	PSMB9->PSMB7->UCHL5->PSMD4->UBC->MDM2->AKT1->RAC1->IQGAP1->CALM1->KCNQ2	41	0.0024
PGRMC1	BCL11A	9.48E-10	1.72E-07	0.430607002	PGRMC1->M6PR->IGF1->IGF1R->ESR1->NCOA2->BCL11A	147	0.00245
CRB3	TFCP2	2.49E-05	0.000738693	-0.304602375	CRB3->PARD6A->CDC42->BNIP2->BCL2->TP53->YY1->TFCP2	33	0.00245
MAP7D1	HMOX1	5.14E-09	7.21E-07	0.413019615	MAP7D1->ARTN->GFRA1->RET->SHC1->EGFR->UBC->HIF1A->HMOX1	116	0.00245
SERPINB2	CD55	9.15E-07	5.21E-05	0.351710447	SERPINB2->PLAU->JUN->MAPK1->GRB2->VAV1->CD19->CR2->C3->CD55	54	0.0025
ANGPTL4	UPP1	4.40E-08	4.34E-06	0.389126254	ANGPTL4->LPL->APOE->APP->PSEN1->CTNNB1->GSK3B->TP53->RRM2B->RRM1->RRM2->TYMS->TK1->CDA->TYMP->UPP1	86	0.0025
MAP7D1	FPR1	1.15E-09	2.02E-07	0.428654767	MAP7D1->ARTN->GFRA1->RET->SHC1->EGFR->UBC->TP53->NPM1->NCL->S100A11->ANXA1->FPR1	148	0.0025
IGKC	TFCP2	1.39E-05	0.000465252	-0.313508073	IGKC->ERBB4->EGF->SRC->ESR1->EP300->YY1->TFCP2	37	0.0025
CDON	KCNQ2	1.46E-06	7.62E-05	0.345484108	CDON->CDH2->CTNNB1->IQGAP1->CALM1->KCNQ2	49	0.0025
CD79A	LTF	1.02E-05	0.000358462	0.31814697	CD79A->BLNK->GRB2->ERBB2->CTNNB1->PSEN1->APP->A2M->LYZ->LTF	43	0.00255
RNF43	GALNT1	2.90E-09	4.49E-07	-0.41909189	RNF43->AKAP8L->DHX9->SF3A2->SNRPD3->LSM7->EXOSC6->CHPF->GALNT1	138	0.00255
CDON	ARHGEF19	8.30E-06	0.000306123	-0.321161988	CDON->CDH2->CTNNB1->DVL1->DAAM1->ARHGEF19	44	0.00255
COL4A5	CGGBP1	6.29E-09	8.60E-07	0.410847353	COL4A5->COL4A3->COL4A4->IGBP1->PPP2CA->SGOL1->PLK1->CDC25C->CHEK2->TP53->MDM2->AKT1->RAC1->NCKAP1->CYFIP1->FMR1->CGGBP1	123	0.0026
PIGR	SPINK5	2.69E-10	6.05E-08	0.443085473	PIGR->CD79A->BLNK->GRB2->ERBB2->CTNNB1->CTNNA1->JUP->DSG1->KLK5->SPINK5	187	0.0026
PSMB9	CCL18	1.50E-07	1.15E-05	0.37454081	PSMB9->PSMB7->UCHL5->PSMD4->UBC->EGFR->GRB2->LCP2->LCK->CD4->CCR5->CCL13->CCR3->CCL18	76	0.0026
IGKC	CD177	2.50E-08	2.68E-06	0.395614534	IGKC->ERBB4->EGF->SRC->ESR1->NCOA3->CREBBP->MYB->PRTN3->CD177	101	0.0026
RNF43	RBM39	3.98E-09	5.96E-07	0.41573387	RNF43->AKAP8L->DHX9->SF3A2->RBM39	130	0.0026
SLC11A1	ESRRG	2.24E-05	0.000677176	-0.306259285	SLC11A1->ZBTB17->MYC->EP300->SIRT1->PPARGC1A->ESRRG	35	0.00265
SLC11A1	CCL18	1.36E-07	1.07E-05	0.375794268	SLC11A1->ZBTB17->MYC->EP300->RELA->NFKB1->CCL5->CCR3->CCL18	80	0.0027
PQBP1	SCML1	8.83E-10	1.65E-07	0.43132396	PQBP1->TXNL4A->CD2BP2->CD2->LCK->NFKBIA->RELA->HDAC1->SIN3A->MECP2->CDKL5->NHS->SCML1	163	0.0027
MAVS	BCL11A	2.23E-07	1.62E-05	0.369694606	MAVS->IRF3->CREBBP->NCOA3->AR->NCOA2->BCL11A	72	0.0027
CCL7	UPP1	1.88E-06	9.30E-05	0.342072139	CCL7->CCR5->CD4->IFNG->IFNGR1->STAT1->EP300->TP53->RRM2B->RRM1->RRM2->TYMS->TK1->CDA->TYMP->UPP1	51	0.00275

CCL7	PIK3AP1	5.54E-07	3.47E-05	0.35823459	CCL7->CCR5->CD4->IFNG->CD80->CD28->PIK3R1->PIK3AP1	61	0.00275
PIGR	CYP2J2	2.93E-07	2.06E-05	0.366304832	PIGR->CD79A->BLNK->GRB2->ERBB2->CTNNB1->RUVBL1->KAT5->PLA2G4A->CYP2J2	71	0.00275
RNF43	MID1	6.00E-10	1.18E-07	0.43518324	RNF43->AKAP8L->DHX9->SF3A2->YBX1->TP53->SP1->SMAD3->RUNX2->SP7->MID1	180	0.00275
RNF43	SLA	2.77E-10	6.19E-08	-0.442797377	RNF43->AKAP8L->DHX9->SF3A2->YBX1->TP53->BRCA1->RBBP8->LMO4->LDB1->LHX3->IFT172->SLA	228	0.00275
IGJ	HLA-DRA	1.68E-07	1.26E-05	0.37319543	IGJ->IL2->STAT3->EP300->CREB1->CIITA->HLA-DRA	79	0.00275
PRF1	FPR1	7.83E-06	0.000290868	0.322012145	PRF1->CD8A->IL2->STAT3->EP300->TP53->NPM1->NCL->S100A11->ANXA1->FPR1	46	0.0028
HLA-DQB2	TNFRSF1B	2.71E-08	2.85E-06	0.394700674	HLA-DQB2->ZFYVE9->SMAD2->EP300->RELA->CHUK->TRAF2->TNFRSF1B	107	0.0028
IGJ	ZAP70	7.88E-07	4.63E-05	0.353659269	IGJ->IL2->LCK->ZAP70	61	0.0028
PSMB9	SLA	1.74E-08	1.99E-06	0.39965776	PSMB9->PSMB7->UCHL5->PSMD4->UBC->TP53->BRCA1->RBBP8->LMO4->LDB1->LHX3->IFT172->SLA	124	0.00285
SYK	ABCB1	8.79E-06	0.000319499	0.320312739	SYK->CBL->EGFR->UBC->TP53->ABCB1	45	0.00285
SLC2A3	A1CF	6.63E-10	1.26E-07	-0.434194713	SLC2A3->SP1->MYC->CTNNB1->PSEN1->APP->APOE->LDLR->APOB->APOBEC1->A1CF	220	0.0029
PTGER1	MTMR3	5.36E-10	1.09E-07	-0.436316723	PTGER1->BDKRB2->KNG1->EGF->SRC->PTK2->PTEN->INPP4B->MTMR3	205	0.0029
SLC22A18	CYP2J2	2.03E-08	2.27E-06	0.397960612	SLC22A18->CDKN1C->CDK4->RB1->TP53->KAT5->PLA2G4A->CYP2J2	118	0.0029
SAV1	BCL11A	1.55E-08	1.82E-06	-0.400935861	SAV1->RASSF1->HRAS->RAF1->HSP90AA1->ESR1->NCOA2->BCL11A	127	0.0029
PTGER1	SOS2	8.16E-07	4.76E-05	-0.353205254	PTGER1->BDKRB2->KNG1->EGF->GRB2->SOS2	65	0.00295
CHST2	CD2	2.63E-10	5.97E-08	0.443282043	CHST2->SELL->CD34->MYB->CREBBP->RELA->NFKBIA->LCK->CD2	258	0.00295
NOP10	C18orf10	6.35E-09	8.63E-07	0.410752447	NOP10->DKC1->RUVBL1->CTNNB1->BTRC->NFKBIA->NFKB1->C18orf10	141	0.00295
RNF43	CD55	2.57E-09	4.04E-07	-0.420360281	RNF43->AKAP8L->DHX9->SF3A2->CD2BP2->CD2->LCK->SYK->CD19->CR2->C3->CD55	154	0.00295
SERPINB2	PIK3AP1	9.96E-07	5.60E-05	0.350590435	SERPINB2->PLAU->JUN->MAPK8->IRS1->PIK3R1->PIK3AP1	62	0.003
RNF43	KLRD1	1.22E-08	1.47E-06	-0.40361282	RNF43->AKAP8L->DHX9->SF3A2->CD2BP2->CD2->LCK->SYK->TYROBP->KLRD1	138	0.003
SATB2	PIK3AP1	1.65E-08	1.90E-06	-0.400284705	SATB2->HOXA2->CD1D->B2M->CD8A->LCK->LCP2->GRB2->PIK3R1->PIK3AP1	130	0.003
SERPINB2	UPP1	5.59E-06	0.000224908	0.32687245	SERPINB2->PLAU->JUN->MAPK8->TP53->RRM2B->RRM1->RRM2->TYMS->TK1->CDA->TYMP->UPP1	50	0.00305
PRF1	ZFHX3	3.18E-06	0.000142113	-0.334872349	PRF1->GZMB->CASP3->BCL2->MYB->ZFHX3	51	0.00305
SLC2A3	HMOX1	9.07E-10	1.67E-07	0.431046743	SLC2A3->SP1->HIF1A->HMOX1	191	0.0031
SATB2	CRYZ	9.28E-09	1.18E-06	-0.406633202	SATB2->HOXA2->CD1D->B2M->CD8A->IL2->STAT3->RAC1->NCF2->DECR1->CRYZ	142	0.0031
CEACAM1	ARHGEF19	1.31E-05	0.000440523	0.314456561	CEACAM1->PTPN11->GRB2->ERBB2->CTNNB1->DVL1->DAAM1->ARHGEF19	45	0.00315
GAS1	FPR1	4.54E-06	0.000190213	0.329840769	GAS1->SHH->FGF8->FGFR1->PLCG1->EGFR->UBC->TP53->NPM1->NCL->S100A11->ANXA1->FPR1	51	0.00315
PRF1	THEMIS	1.10E-09	1.96E-07	0.429092498	PRF1->CD8A->IL2->THEMIS	190	0.00315
NOP10	BCL11A	6.51E-07	3.94E-05	-0.356140856	NOP10->DKC1->RUVBL1->CTNNB1->AR->NCOA2->BCL11A	73	0.00315
PSMB9	APBB1IP	3.49E-06	0.000152772	0.33354619	PSMB9->PSMB7->UCHL5->PSMD4->UBC->EGFR->SRC->PTK2->TLN1->APBB1IP	54	0.00315
SYK	ETV6	1.86E-05	0.000585892	0.309072401	SYK->CBL->SHC1->JAK2->ETV6	43	0.00315
RNF43	CD69	3.01E-08	3.10E-06	-0.393472713	RNF43->AKAP8L->DHX9->SF3A2->CD2BP2->CD2->LCK->IL2->CD69	126	0.00315
SLC11A1	BCL11A	6.72E-07	4.03E-05	-0.355745355	SLC11A1->ZBTB17->MYC->ESR1->NCOA2->BCL11A	75	0.0032
GAS1	CYP2J2	6.39E-07	3.88E-05	-0.356383733	GAS1->SHH->FGF8->FGFR1->PLCG1->EGFR->UBC->TP53->KAT5->PLA2G4A->CYP2J2	75	0.0032
IGHD	SLA	4.56E-09	6.63E-07	0.414302207	IGHD->GHRH->GCG->LEP->STAT3->EP300->MYOD1->TCF3->TAL1->LMO2->LDB1->LHX3->IFT172->SLA	160	0.0032

CEACAM1	FPR1	7.88E-06	0.000292549	-0.321910795	CEACAM1->PTPN11->EGFR->UBC->TP53->NPM1->NCL->S100A11->ANXA1->FPR1	51	0.00325
GAS1	HAS2	1.91E-08	2.17E-06	0.398608935	GAS1->SHH->NOG->BMP2->HAS2	140	0.00325
SERPINB2	ARHGEF19	2.75E-05	0.000796636	-0.30309113	SERPINB2->PLAU->NFKB1->NFKBIA->BTRC->CTNNB1->DVL1->DAAM1->ARHGEF19	40	0.00325
SERPINB2	NT5E	6.61E-06	0.00025608	0.324477614	SERPINB2->PLAU->JUN->MAPK8->TP53->USP7->GMPS->IMPDH2->NT5E	52	0.00325
MAP7D1	CD69	3.52E-08	3.56E-06	0.391681182	MAP7D1->ARTN->GFRA1->RET->SHC1->IL2->CD69	128	0.00325
MMP8	CCL18	5.34E-09	7.43E-07	0.412604679	MMP8->TIMP2->MMP2->JUN->IL2->CD8A->CD4->CCR5->CCL13->CCR3->CCL18	166	0.0033
ANGPTL4	GNA11	1.53E-06	7.90E-05	-0.344863457	ANGPTL4->LPL->APOE->APP->PSEN1->CTNNB1->ERBB2->GRB2->RET->GDNF->EDNRB->GNA11	67	0.0033
ANGPTL4	SHANK2	2.31E-06	0.000109451	-0.339297883	ANGPTL4->LPL->APOE->APP->PSEN1->CTNNB1->CCND1->CDKN1B->AKT1->MTOR->FKBP1A->RYR1->HOMER1->SHANK2	61	0.0033
SALL2	SMAD7	3.58E-05	0.000979496	0.298906045	SALL2->NGFR->SHC1->ERBB2->CTNNB1->SMAD7	39	0.0033
GAS1	GALNT1	5.58E-06	0.000224569	0.326912518	GAS1->SHH->FGF8->FGFR1->FRS2->GRB2->LCP2->LCK->CD2->CD2BP2->SF3A2->SNRPE->LSM4->EXOSC10->CHPF->GALNT1	58	0.00335
GAS1	SCML1	3.30E-08	3.38E-06	-0.392415629	GAS1->SHH->FGF8->FGFR1->PLCG1->EGFR->UBC->TP53->HDAC1->SIN3A->MECP2->CDKL5->NHS->SCML1	139	0.00335
ANGPTL4	FZD8	1.55E-05	0.000509266	-0.311829266	ANGPTL4->LPL->APOE->APP->PSEN1->CTNNB1->AXIN1->LRP6->FZD8	45	0.00335
MAP7D1	A1CF	2.19E-08	2.40E-06	-0.397108707	MAP7D1->ARTN->GFRA1->RET->GRB2->ERBB2->CTNNB1->PSEN1->APP->APOE->LDLR->APOB->APOBEC1->A1CF	143	0.00335
CASP1	IDH2	7.48E-06	0.00028325	0.322678021	CASP1->IL18->IFNG->IL2->STAT3->SRC->CTNNB1->PITX2->SUCLG1->SDHB->FH->MDH2->CS->ACO2->IDH2	54	0.00335
SALL2	HMOX1	2.93E-05	0.000841524	0.302060706	SALL2->NGFR->SHC1->EGFR->UBC->HIF1A->HMOX1	41	0.0034
SLC2A3	PIK3AP1	1.15E-09	2.02E-07	0.428643966	SLC2A3->SP1->ESR1->IGF1R->PIK3R1->PIK3AP1	242	0.00345
APOBEC1	KLRD1	1.05E-05	0.00036853	-0.317637598	APOBEC1->APOB->LDLR->APOE->APP->PSEN1->CTNNB1->ERBB2->GRB2->VAV1->SYK->TYROBP->KLRD1	51	0.00345
PIGR	PTPRH	4.08E-06	0.000173089	0.331365798	PIGR->CD79A->BLNK->GRB2->MAPK1->ELK1->SRF->PTPRH	60	0.00345
C18orf1	SMAD7	6.32E-08	5.81E-06	0.384899701	C18orf1->TGFBI->FN1->TGFB1->TGFBR1->SMAD7	118	0.00345
SERPINB2	FCGR3B	4.57E-06	0.000190801	0.329740185	SERPINB2->PLAU->JUN->MAPK8->IRS1->PIK3R1->CBL->SYK->FCER1G->FCGR3B	59	0.0035
PTGER3	MARCKS	3.39E-06	0.000149655	0.333972228	PTGER3->KNG1->EGF->SRC->GJA1->PRKCA->MARCKS	61	0.0035
PSMB9	PIK3AP1	3.05E-06	0.000137774	0.335429183	PSMB9->PSMB7->UCHL5->PSMD4->UBC->EGFR->GRB2->PIK3R1->PIK3AP1	61	0.0035
HSPB1	ATP6V1E1	1.56E-07	1.19E-05	0.374080781	HSPB1->MAPKAPK2->MAPK14->CREB1->EP300->RELA->CHUK->TRAF2->TNFRSF11A->TNFSF11->TCIRG1->ATP6V1B1->ATP6V1D->ATP6V1A->ATP6V1E1	97	0.00355
SERPINB2	MID1	1.06E-06	5.86E-05	-0.34978638	SERPINB2->PLAU->JUN->RUNX2->SP7->MID1	77	0.00355
NOP10	FCGR3B	6.15E-06	0.000242377	0.325505349	NOP10->DKC1->TERT->AKT1->RAC1->VAV1->SYK->FCER1G->FCGR3B	59	0.00355
NOP10	SUV420H1	8.79E-08	7.45E-06	-0.380994186	NOP10->DKC1->RUVBL1->CTNNB1->AR->NCOA2->SUV420H1	110	0.0036
FCRL5	HLA-DRA	1.14E-07	9.20E-06	0.377903932	FCRL5->BCL9->CTNNB1->CCND1->CREB1->CIITA->HLA-DRA	106	0.0036
MAP7D1	GFPT1	6.72E-09	9.02E-07	-0.410142671	MAP7D1->ARTN->GFRA1->RET->SHC1->EGFR->UBC->TP53->ATF3->ATF4->ASNS->GLUL->GFPT1	169	0.0036
SLC11A1	SLA	6.49E-08	5.92E-06	0.384574921	SLC11A1->ZBTB17->MYC->SP1->HDAC1->TAL1->LMO2->LDB1->LHX3->IFT172->SLA	128	0.00365
GAS1	ABCB1	1.03E-05	0.000361709	-0.317964492	GAS1->SHH->FGF8->FGFR1->PLCG1->EGFR->UBC->TP53->ABCB1	55	0.00365
ANGPTL4	BCL11A	1.16E-07	9.36E-06	-0.377625227	ANGPTL4->LPL->APOE->APP->PSEN1->CTNNB1->AR->NCOA2->BCL11A	107	0.00365
PITX1	LPHN2	5.90E-08	5.53E-06	-0.385694506	PITX1->LHB->LHCGR->ARRB2->MAPK1->MYC->EP300->SMAD2->TGFBR1->FKBP1A->RYR1->HOMER1->SHANK2->LPHN2	132	0.00365

EEF1B2	KCNQ2	3.38E-07	2.33E-05	0.36450214	EEF1B2->RPL6->RPL5->MDM2->AKT1->RAC1->IQGAP1->CALM1->KCNQ2	88	0.0037
NFIB	HMOX1	9.84E-06	0.000349017	-0.318658126	NFIB->HNF1A->HNF4A->SMAD4->SMAD2->EP300->HIF1A->HMOX1	58	0.0037
SLC11A1	SUV420H1	1.00E-07	8.31E-06	-0.379434349	SLC11A1->ZBTB17->MYC->ESR1->NCOA2->SUV420H1	112	0.00375
IGHD	PIK3AP1	2.34E-07	1.69E-05	0.369109163	IGHD->GHRH->GCG->LEP->STAT3->JAK2->SHC1->PIK3R1->PIK3AP1	97	0.00375
RNF43	SHANK2	8.01E-08	6.96E-06	0.382101602	RNF43->AKAP8L->DHX9->SF3A2->CD2BP2->CD2->LCK->LCP2->VAV1->RAC1->AKT1->MTOR->FKBP1A->RYR1->HOMER1->SHANK2	128	0.00375
COL4A5	CASP9	2.34E-05	0.00070028	0.305562692	COL4A5->COL4A3->COL4A4->IGBP1->PPP2CA->SGOL1->PLK1->CDC25C->CHEK2->TP53->APAF1->CASP9	46	0.00385
COL4A5	KCNQ2	1.25E-06	6.75E-05	0.347534523	COL4A5->COL4A3->COL4A4->IGBP1->PPP2CA->SGOL1->PLK1->CDC25C->CHEK2->TP53->EP300->CREB1->CAMK2B->CALM1->KCNQ2	79	0.00385
MAVS	SLA	9.12E-08	7.67E-06	-0.380553125	MAVS->IRF3->CREBBP->TP53->BRCA1->RBBP8->LMO4->LDB1->LHX3->IFT172->SLA	125	0.0039
CCL7	SLA	1.06E-07	8.66E-06	0.378714623	CCL7->CCR1->CCL5->NFKB1->RELA->HDAC1->TAL1->LMO2->LDB1->LHX3->IFT172->SLA	126	0.00395
PTGER1	RASGRP2	1.01E-07	8.33E-06	0.379316859	PTGER1->BDKRB2->KNG1->EGF->EGFR->SOS1->HRAS->RAF1->KRAS->RASGRP2	126	0.00395
CXCL14	C18orf10	1.79E-09	2.97E-07	-0.424136403	CXCL14->CXCR4->CD4->LCK->NFKBIA->NFKB1->C18orf10	275	0.00395
IGKC	HLA-DRA	1.70E-05	0.000543441	0.31048081	IGKC->ERBB4->EGF->SRC->ESR1->EP300->CREB1->CIITA->HLA-DRA	50	0.00395
PGRMC1	UPP1	1.14E-07	9.20E-06	-0.377935065	PGRMC1->M6PR->IGF1->IRS1->MAPK8->TP53->RRM2B->RRM1->RRM2->TYMS->TK1->CDA->TYMP->UPP1	126	0.004
CYP4F2	JAK2	1.98E-06	9.70E-05	-0.341347671	CYP4F2->PTGS2->IL6->STAT3->JAK2	78	0.00405
FCRL5	THEMIS	3.16E-09	4.83E-07	0.418170204	FCRL5->BCL9->CTNNB1->CCND1->STAT3->IL2->THEMIS	272	0.0041
CASP1	RASGEF1B	9.27E-09	1.18E-06	0.406641905	CASP1->IL18->IFNG->IL2->STAT3->RAC1->ARHGDIA->RHOA->ROCK2->PPP1R12A->PRKG1->MRVI1->PRKG2->RASGEF1B	179	0.0041
SATB2	SCML1	8.21E-09	1.07E-06	0.407971543	SATB2->HOXA2->CD1D->B2M->CD8A->IL2->STAT3->EP300->TP53->HDAC1->SIN3A->MECP2->CDKL5->NHS->SCML1	201	0.0041
ANGPTL4	CRIM1	7.71E-08	6.79E-06	-0.382547341	ANGPTL4->LPL->APOE->APP->PSEN1->CTNNB1->SMAD4->SMAD1->BMPR1A->BMP4->CRIM1	141	0.00415
NOP10	CCL18	1.89E-06	9.33E-05	0.341987632	NOP10->DKC1->RUVBL1->CTNNB1->BTRC->NFKBIA->NFKB1->CCL5->CCR3->CCL18	79	0.00415
RNF43	ENPP3	7.10E-09	9.45E-07	0.40954811	RNF43->AKAP8L->DHX9->SF3A2->CD2BP2->CD2->LCK->IL2->IL5->IL3->ENPP3	208	0.00415
NFIB	FCGR3B	2.71E-07	1.94E-05	-0.367260031	NFIB->HNF1A->HNF4A->SMAD4->SMAD2->EP300->STAT3->EGFR->CBL->SYK->FCER1G->FCGR3B	105	0.0042
SLC2A3	CXCL13	1.24E-08	1.48E-06	0.403423543	SLC2A3->SP1->EP300->STAT1->CXCL9->CXCR3->CXCL13	199	0.00425
CEACAM1	SPINK5	1.76E-08	2.00E-06	0.399540856	CEACAM1->PTPN11->GRB2->ERBB2->CTNNB1->CDH1->JUP->DSG1->KLK5->SPINK5	192	0.00425
CCL7	CRIM1	3.11E-07	2.17E-05	-0.365556417	CCL7->CCR5->CD4->IFNG->IFNGR1->STAT1->EP300->SMAD2->SMAD4->SMAD1->BMPR1A->BMP4->CRIM1	105	0.00425
NOP10	CTSA	6.07E-06	0.000239818	-0.325694638	NOP10->DKC1->TERT->AKT1->TSC2->YWHAZ->GP1BA->F2->GLA->GLB1->CTSA	68	0.00425
PIGR	CD177	1.97E-06	9.62E-05	0.341483242	PIGR->CD79A->BLNK->GRB2->MAPK1->JUN->SPI1->PRTN3->CD177	80	0.00425
MAP7D1	UPP1	5.75E-08	5.41E-06	0.385993048	MAP7D1->ARTN->GFRA1->RET->SHC1->EGFR->UBC->TP53->RRM2B->RRM1->RRM2->TYMS->TK1->CDA->TYMP->UPP1	154	0.00425
MAVS	GFPT1	1.09E-05	0.000378441	0.317161671	MAVS->IRF3->CREBBP->TP53->ATF3->ATF4->ASNS->GLUL->GFPT1	64	0.00425
SLC2A3	PIK3CG	1.93E-08	2.17E-06	0.398540596	SLC2A3->SP1->ESR1->AKT1->PIK3CG	193	0.0043
PGRMC1	FPR1	1.76E-07	1.31E-05	-0.372632759	PGRMC1->M6PR->IGF1->IRS1->MAPK8->TP53->NPM1->NCL->S100A11->ANXA1->FPR1	120	0.0043
PLEKHB1	KCNQ2	4.06E-09	6.01E-07	0.415546597	PLEKHB1->SELPLG->SELE->NFKB1->RELA->EP300->CREB1->CAMK2B->CALM1->KCNQ2	298	0.0043

ZC3H12A	ASS1	3.68E-05	0.000997589	0.298463881	ZC3H12A->CCL2->CCR5->CD4->LCK->LCP2->VAV1->RAC1->AKT1->NOS3->ARG2->OTC->ASS1	45	0.0043
MAP7D1	KANK4	7.75E-09	1.01E-06	0.408587537	MAP7D1->ARTN->GFRA1->RET->SHC1->EGFR->UBC->TP53->TBP->POLR2A->POLR2H->POLR3D->POLR3F->POLR3C->KANK4	233	0.0043
CDON	PURB	1.15E-06	6.28E-05	-0.348687667	CDON->CDH2->CTNNB1->SRC->VCL->TPM1->TNNT2->MYBPC3->MYH6->PURB	86	0.0043
IGJ	CD177	1.49E-07	1.15E-05	0.37463569	IGJ->IL2->JUN->SPI1->PRTN3->CD177	129	0.00435
SERPINB2	MYEF2	2.83E-05	0.000817135	-0.302607847	SERPINB2->PLAU->NFKB1->RELA->HDAC1->SIN3A->HDAC9->MYEF2	48	0.0044
CXCL14	JAK2	8.99E-08	7.58E-06	-0.380728109	CXCL14->CXCR4->CD4->LCK->PTPN6->JAK2	143	0.0044
IGHD	ACCN2	2.00E-05	0.000618861	-0.307975304	IGHD->GHRH->GCG->LEP->STAT3->SRC->GJA1->PRKCA->PICK1->ACCN2	58	0.0044
CEACAM1	GFPT1	5.60E-06	0.000225004	0.326847894	CEACAM1->PTPN11->IRS1->MAPK8->JUN->ATF3->ATF4->ASNS->GLUL->GFPT1	72	0.00445
PTGER1	RUNDC3A	5.19E-08	4.96E-06	-0.387202742	PTGER1->BDKRB2->KNG1->EGF->EGFR->NCK1->TNIK->RAP2A->RUNDC3A	165	0.00445
RB1CC1	UPP1	6.36E-06	0.000247997	-0.325030505	RB1CC1->PTK2->PTEN->TP53->RRM2B->RRM1->RRM2->TYMS->TK1->CDA->TYMP->UPP1	71	0.0045
PTGER1	HMOX1	7.42E-06	0.000281735	0.322791307	PTGER1->BDKRB2->KNG1->EGF->EGFR->UBC->HIF1A->HMOX1	70	0.0045
VIP	SPINK5	1.68E-08	1.93E-06	0.400085759	VIP->VIPR1->ADCYAP1->ADCYAP1R1->GCG->LEP->STAT3->SRC->CTNNB1->CDH5->JUP->DSG1->KLK5->SPINK5	213	0.0045
MAP7D1	CCL18	3.36E-08	3.42E-06	0.392234765	MAP7D1->ARTN->GFRA1->RET->GRB2->LCP2->LCK->CD4->CCR5->CCL13->CCR3->CCL18	178	0.00455
MAP7D1	KLRD1	1.39E-07	1.09E-05	0.3755209	MAP7D1->ARTN->GFRA1->RET->GRB2->VAV1->SYK->TYROBP->KLRD1	139	0.00455
APOBEC1	ETV6	1.83E-05	0.000577808	0.309315233	APOBEC1->APOB->LDLR->APOE->APP->PSEN1->CTNNB1->ERBB2->GRB2->JAK2->ETV6	61	0.0046
EEF1B2	MARCKS	1.79E-05	0.000566706	-0.309672663	EEF1B2->RPL6->RPL5->MDM2->AKT1->RAC1->PAK1->CDC42->PLD1->PRKCA->MARCKS	61	0.0046
LEPRE1	KANK4	1.01E-07	8.33E-06	0.379358448	LEPRE1->PPIB->BSG->MMP1->JUN->MAPK8->TP53->TBP->POLR2A->POLR2H->POLR3D->POLR3F->POLR3C->KANK4	147	0.0046
CHST2	KLRD1	8.32E-09	1.08E-06	0.407819429	CHST2->SELL->CD34->MYB->CREBBP->TP53->UBC->EGFR->CBL->SYK->TYROBP->KLRD1	285	0.0046
GAS1	ST3GAL1	9.29E-08	7.79E-06	0.380337648	GAS1->SHH->FGF8->FGFR2->FGF23->GALNT3->GCNT1->ST3GAL1	152	0.00465
GAS1	GFPT1	7.76E-06	0.000289707	-0.322147554	GAS1->SHH->FGF8->FGFR1->PLCG1->EGFR->UBC->TP53->ATF3->ATF4->ASNS->GLUL->GFPT1	72	0.00465
SERPINB2	CCL18	5.67E-06	0.000227478	0.326672778	SERPINB2->PLAU->NFKB1->CCL5->CCR3->CCL18	75	0.00465
FCRL5	ZAP70	1.28E-06	6.86E-05	0.347281181	FCRL5->BCL9->CTNNB1->ERBB2->GRB2->LCP2->ZAP70	88	0.00465
C19orf61	MARCKS	6.56E-06	0.000254633	-0.324581454	C19orf61->UPF1->UPF2->LSM1->LSM7->SNRPD3->SF3A2->CD2BP2->CD2->LCK->LCP2->GRB2->WASL->CDC42->PLD1->PRKCA->MARCKS	74	0.0047
LEPRE1	FPR1	1.98E-05	0.000615484	0.308131483	LEPRE1->PPIB->BSG->MMP1->JUN->MAPK8->TP53->NPM1->NCL->S100A11->ANXA1->FPR1	62	0.00475
CHST2	CXCL13	9.29E-09	1.18E-06	0.406622037	CHST2->SELL->CD34->MYB->CREBBP->RELA->NFKB1->CCL5->CCR3->CCL11->CXCR3->CXCL13	267	0.00475
SLC11A1	CLEC4E	1.14E-08	1.39E-06	0.404334566	SLC11A1->ZBTB17->MYC->EP300->RELA->IKBKB->BCL10->CARD9->CLEC4E	270	0.0048
CXCL14	A1CF	4.62E-08	4.53E-06	0.388543355	CXCL14->CXCR4->CD4->LCK->LCP2->GRB2->ERBB2->CTNNB1->PSEN1->APP->APOE->LDLR->APOB->APOBEC1->A1CF	177	0.00485
SLC11A1	UPP1	3.24E-05	0.000903817	0.300500114	SLC11A1->ZBTB17->MYC->EP300->TP53->RRM2B->RRM1->RRM2->TYMS->TK1->CDA->TYMP->UPP1	53	0.0049
HSPB1	IDH2	3.06E-05	0.000868814	-0.301402532	HSPB1->MAPKAPK2->MAPK14->CREB1->CCND1->CTNNB1->PITX2->SUCLG1->SDHB->FH->MDH2->CS->ACO2->IDH2	53	0.0049
GADD45GIP1	COPA	2.76E-06	0.000126612	-0.336797846	GADD45GIP1->GADD45G->PCNA->CCND1->CTNNB1->SRC->GNB2L1->PRKCE->COPB2->COPB1->COPA	83	0.0049
PIGR	IDH2	2.72E-05	0.000791871	0.303228246	PIGR->CD79A->BLNK->GRB2->ERBB2->CTNNB1->PITX2->SUCLG1->SDHB->FH->MDH2->CS->ACO2->IDH2	55	0.0049

CD79A	RASGRP2	1.73E-05	0.000549464	0.310220262	CD79A->BLNK->GRB2->PIK3R1->PIK3CA->KRAS->RASGRP2	66	0.00505
ANGPTL4	GFPT1	1.14E-06	6.23E-05	-0.348843917	ANGPTL4->LPL->APOE->APP->PSEN1->CTNNB1->GSK3B->TP53->ATF3->ATF4->ASNS->GLUL->GFPT1	101	0.00505
AKR1C3	ESRRG	2.42E-05	0.000721475	0.305027128	AKR1C3->PTGDS->PTGS2->IL6->STAT3->EP300->SIRT1->PPARGC1A->ESRRG	62	0.00505
SERPINB2	CRIM1	9.84E-07	5.56E-05	-0.350751563	SERPINB2->PLAU->JUN->SMAD4->SMAD1->BMPR1A->BMP4->CRIM1	104	0.0051
HLA-DQB2	CD2	5.56E-07	3.48E-05	0.358177996	HLA-DQB2->ZFYVE9->SMAD2->EP300->STAT3->IL2->LCK->CD2	109	0.0051
MAVS	CLEC4E	2.27E-08	2.48E-06	-0.39666576	MAVS->IRF3->CREBBP->RELA->IKBKB->BCL10->CARD9->CLEC4E	266	0.0051
MAVS	PIK3AP1	2.06E-05	0.000632555	-0.307548699	MAVS->IRF3->CREBBP->TP53->MAPK8->IRS1->PIK3R1->PIK3AP1	63	0.0051
RB1CC1	SLITRK6	2.52E-08	2.69E-06	-0.395527871	RB1CC1->PTK2->GRB2->NTRK1->SLITRK6	259	0.00515
PTGER1	PURB	2.84E-07	2.01E-05	-0.366683023	PTGER1->BDKRB2->KNG1->EGF->SRC->VCL->TPM1->TNNT2->MYBPC3->MYH6->PURB	134	0.00515
CXCL14	SLC25A11	2.97E-08	3.08E-06	-0.393633577	CXCL14->CXCR4->CD4->IFNG->IL2->STAT3->EP300->TP53->BCL2->BNIP3->BCKDHB->DLD->OGDH->SLC25A11	250	0.00515
FCRL5	SLA	4.50E-08	4.43E-06	0.388859956	FCRL5->BCL9->CTNNB1->TCF4->TAL1->LMO2->LDB1->LHX3->IFT172->SLA	203	0.00515
IGKC	CLEC2D	1.98E-08	2.22E-06	0.398235809	IGKC->ERBB4->EGF->EGFR->UBC->RIPK1->TRAF2->TNFRSF11A->TNFSF11->CLEC2D	294	0.00515
NOP10	VNN2	9.00E-08	7.58E-06	0.380717677	NOP10->DKC1->RUVBL1->CTNNB1->BTRC->NFKBIA->NFKB1->ICAM1->ITGB2->VNN2	170	0.0052
PARVA	KANK4	4.13E-07	2.73E-05	0.361977094	PARVA->ILK->AKT1->MDM2->TP53->TBP->POLR2A->POLR2H->POLR3D->POLR3F->POLR3C->KANK4	127	0.0052
RNF43	HMOX1	4.81E-07	3.09E-05	-0.360033968	RNF43->AKAP8L->DHX9->SF3A2->YBX1->TP53->HIF1A->HMOX1	115	0.0052
CDON	RASGRP2	1.02E-05	0.000358379	0.318167154	CDON->SHH->FGF8->FGFR1->FRS2->PTPN11->KRAS->RASGRP2	77	0.00525
PITX1	MARCKS	2.49E-05	0.000738711	-0.304587286	PITX1->LHB->LHCGR->ARRB2->MAPK1->GRB2->WASL->CDC42->PLD1->PRKCA->MARCKS	63	0.00525
MAP7D1	TFCP2	5.02E-07	3.20E-05	-0.359506183	MAP7D1->ARTN->GFRA1->RET->SHC1->EGFR->STAT3->EP300->YY1->TFCP2	118	0.0053
HSPB1	NUPR1	4.89E-06	0.000200576	0.328804581	HSPB1->MAPKAPK2->MAPK14->ATF2->MAPK8->TP53->NUPR1	83	0.0054
MAGT1	FCGR3B	6.22E-08	5.77E-06	-0.385084259	MAGT1->DDOST->SSR4->RPS5->RPL5->MDM2->AKT1->RAC1->VAV1->SYK->FCER1G->FCGR3B	203	0.0054
CD79A	CD177	1.29E-05	0.000435268	0.314652442	CD79A->BLNK->GRB2->MAPK1->JUN->SPI1->PRTN3->CD177	76	0.0055
ANGPTL4	ESRRG	2.09E-05	0.000638861	-0.307321	ANGPTL4->LPL->APOE->APP->PSEN1->CTNNB1->GSK3B->TP53->SIRT1->PPARGC1A->ESRRG	68	0.0055
VIP	RPRM	1.92E-06	9.43E-05	0.341771094	VIP->VIPR1->ADCYAP1->POMC->NR4A1->BCL2->TP53->RPRM	101	0.0055
PSMB9	CLEC4E	3.90E-08	3.90E-06	0.390514114	PSMB9->PSMB7->UCHL5->PSMD4->UBC->IKBKG->BCL10->CARD9->CLEC4E	266	0.00555
CD79A	CLEC2D	4.10E-08	4.07E-06	0.389933614	CD79A->BLNK->GRB2->EGFR->UBC->RIPK1->TRAF2->TNFRSF11A->TNFSF11->CLEC2D	270	0.0056
SATB2	ARHGEF19	1.38E-06	7.30E-05	0.346225502	SATB2->HOXA2->CD1D->B2M->CD8A->IL2->STAT3->SRC->CTNNB1->DVL1->DAAM1->ARHGEF19	108	0.00565
PTGER1	SNAPIN	3.77E-06	0.000161877	0.332481584	PTGER1->BDKRB2->KNG1->EGF->EGFR->PLCG1->PDGFRB->SLC9A3R1->CFTR->STX1A->SNAP25->SNAPIN	87	0.00575
SERPINB2	CRYZ	1.55E-05	0.000509266	0.311826181	SERPINB2->PLAU->JUN->IL2->STAT3->RAC1->NCF2->DECR1->CRYZ	76	0.0058
CHST2	TNFRSF1B	5.64E-08	5.33E-06	0.386232005	CHST2->SELL->CD34->MYB->CREBBP->RELA->CHUK->TRAF2->TNFRSF1B	255	0.00585
MAP7D1	FCGR3B	2.35E-07	1.69E-05	0.369058127	MAP7D1->ARTN->GFRA1->RET->GRB2->VAV1->SYK->FCER1G->FCGR3B	159	0.00585
MBNL2	ITGA6	1.54E-06	7.93E-05	0.344796627	MBNL2->CCT3->PPP2CA->SGOL1->PLK1->CDC25C->CHEK2->TP53->PTEN->PTK2->PXN->ITGA4->ITGB1->ITGA6	110	0.00585
PGRMC1	VNN2	6.43E-08	5.88E-06	-0.384692931	PGRMC1->M6PR->PLIN3->IGF2R->PLAUR->PLG->FN1->ITGB1->ITGA8->ITGB2->VNN2	243	0.0059
PTGER1	LMBR1L	9.88E-07	5.57E-05	0.350691779	PTGER1->BDKRB2->KNG1->KLKB1->A2M->LYZ->LCN1-	114	0.0059

					>LMBR1L		
CASP1	CLEC2D	5.48E-08	5.19E-06	0.386571791	CASP1->IL18->IL1B->NFKB1->CHUK->TRAF2->TNFRSF11A->TNFSF11->CLEC2D	270	0.0059
IGKC	TOX2	7.24E-08	6.47E-06	0.383285204	IGKC->ERBB4->EGF->EGFR->UBC->TP53->BCL2->NR4A1->ZNF461->TOX2	231	0.00595
RPS2	CTSA	3.24E-05	0.000904596	-0.300470286	RPS2->RPS3->RPL5->MDM2->AKT1->TSC2->YWHAZ->GP1BA->F2->GLA->GLB1->CTSA	68	0.006
SLC2A3	CYP2J2	7.52E-08	6.64E-06	-0.382845305	SLC2A3->SP1->TP53->KAT5->PLA2G4A->CYP2J2	246	0.00605
PGRMC1	TFCP2	3.81E-06	0.000163285	0.332340611	PGRMC1->M6PR->IGF1->IGF1R->ESR1->EP300->YY1->TFCP2	90	0.00605
RABGEF1	RASGRP2	2.35E-05	0.000703186	-0.305483827	RABGEF1->RABEP1->RAB5A->EGFR->SOS1->HRAS->RAF1->KRAS->RASGRP2	72	0.0061
SAV1	MYEF2	2.69E-06	0.00012368	-0.337162482	SAV1->RASSF1->HRAS->PIK3CA->PTEN->TP53->HDAC1->SIN3A->HDAC9->MYEF2	103	0.0061
SLC2A3	JAK2	1.44E-07	1.12E-05	0.37507784	SLC2A3->SP1->EP300->STAT1->JAK2	187	0.00615
SOX11	HMOX1	6.60E-07	3.97E-05	0.355964228	SOX11->BNIP3->BCL2->TP53->HIF1A->HMOX1	136	0.00615
SOX11	KANK4	7.33E-08	6.53E-06	0.383146141	SOX11->BNIP3->BCL2->TP53->TBP->POLR2A->POLR2H->POLR3D->POLR3F->POLR3C->KANK4	252	0.00615
CRB3	BCL11A	4.97E-06	0.000203294	-0.328575624	CRB3->PARD6A->CDC42->IQGAP1->RAC1->AKT1->ESR1->NCOA2->BCL11A	91	0.00615
ATP2B4	ATP6V1E2	1.21E-06	6.52E-05	-0.348063834	ATP2B4->CALM1->CAMK2B->CREB1->CREBBP->RELA->CHUK->TRAF2->TNFRSF11A->TNFSF11->TCIRG1->ATP6V1B1->ATP6V1D->ATP6V1A->ATP6V1E2	122	0.0062
MAVS	SUV420H1	2.14E-06	0.000102784	0.340344408	MAVS->IRF3->CREBBP->NCOA3->AR->NCOA2->SUV420H1	109	0.0062
APOBEC1	SP5	5.79E-06	0.000230376	0.32638113	APOBEC1->APOB->LDLR->APOE->APP->PSEN1->CTNNB1->SP5	91	0.00625
TET3	SNAPIN	3.35E-07	2.31E-05	-0.364633868	TET3->NANOG->KLF4->CTNNB1->ERBB2->GRB2->PDGFRB->SLC9A3R1->CFTR->STX1A->SNAP25->SNAPIN	163	0.00625
SATB2	CRIM1	1.89E-07	1.40E-05	0.371727531	SATB2->HOXA2->CD1D->B2M->CD8A->IL2->STAT3->EP300->SMAD2->SMAD4->SMAD1->BMPR1A->BMP4->CRIM1	173	0.00625
CHRNA3	ARHGEF19	2.70E-05	0.000788308	0.303342166	CHRNA3->PSMA4->UCHL5->PSMD4->UBC->TP53->GSK3B->CTNNB1->DVL1->DAAM1->ARHGEF19	71	0.0063
RNF43	CCL18	2.06E-07	1.52E-05	-0.37065317	RNF43->AKAP8L->DHX9->SF3A2->CD2BP2->CD2->LCK->CD4->CCR5->CCL5->CCR3->CCL18	175	0.0063
CXCL14	CRYZ	1.35E-07	1.07E-05	-0.375846494	CXCL14->CXCR4->CD4->LCK->LCP2->VAV1->RAC1->NCF2->DECR1->CRYZ	209	0.00635
PGRMC1	CD2	1.07E-05	0.000374305	-0.317374151	PGRMC1->M6PR->IGF1->IRS1->GRB2->LCP2->LCK->CD2	86	0.0064
ANGPTL4	CCL18	1.88E-06	9.30E-05	0.342080948	ANGPTL4->LPL->APOE->APP->PSEN1->CTNNB1->BTRC->NFKBIA->NFKB1->CCL5->CCR3->CCL18	113	0.0064
NOP10	DYNC1I2	2.54E-05	0.000749668	-0.304312472	NOP10->DKC1->RUVBL1->KAT5->TP53->CHEK1->CDC25C->PLK1->NUDC->PAFAH1B1->DCTN1->DCTN2->DYNC1I2	74	0.0064
IGHD	APBB1IP	8.33E-06	0.000306797	0.321112596	IGHD->GHRH->GCG->LEP->STAT3->SRC->PTK2->TLN1->APBB1IP	89	0.0064
PQBP1	BCL11A	3.23E-06	0.000143955	0.334653023	PQBP1->TXNL4A->CD2BP2->CD2->LCK->ZAP70->SHC1->IGF1R->ESR1->NCOA2->BCL11A	104	0.00645
PRF1	CLEC2D	8.31E-08	7.11E-06	0.381660275	PRF1->CD8A->LCK->NFKBIA->CHUK->TRAF2->TNFRSF11A->TNFSF11->CLEC2D	273	0.0065
FCRL5	KLRD1	1.74E-06	8.81E-05	0.343106475	FCRL5->BCL9->CTNNB1->ERBB2->EGFR->CBL->SYK->TYROBP->KLRD1	117	0.0065
GAS1	NUPR1	7.82E-06	0.000290859	0.322029935	GAS1->SHH->FGF8->FGFR1->PLCG1->EGFR->UBC->TP53->NUPR1	92	0.00655
ANGPTL4	SUV420H1	7.78E-07	4.58E-05	-0.3538353	ANGPTL4->LPL->APOE->APP->PSEN1->CTNNB1->AR->NCOA2->SUV420H1	144	0.00665
PRF1	VNN2	3.64E-07	2.46E-05	0.363594006	PRF1->CD8A->IL2->STAT3->IL6->ICAM1->ITGB2->VNN2	168	0.00665
RNF43	HLA-DRA	1.49E-06	7.73E-05	-0.345245597	RNF43->AKAP8L->DHX9->SF3A2->YBX1->TP53->EP300->CREB1->CIITA->HLA-DRA	130	0.0067
CD79A	THEMIS	2.09E-07	1.53E-05	0.370493018	CD79A->SYK->LCK->IL2->THEMIS	190	0.00675
ANGPTL4	ABCB1	1.46E-05	0.000484768	-0.312745377	ANGPTL4->LPL->APOE->APP->PSEN1->CTNNB1->GSK3B->TP53->ABCB1	84	0.00675

MAP7D1	APBB1IP	5.91E-07	3.65E-05	0.357403116	MAP7D1->ARTN->GFRA1->RET->GRB2->PTK2->TLN1->APBB1IP	156	0.00675
NFIB	MID1	2.15E-06	0.000102784	0.340283992	NFIB->HNF1A->HNF4A->SMAD4->SMAD1->RUNX2->SP7->MID1	119	0.00675
IGKC	THEMIS	1.73E-07	1.29E-05	0.372824575	IGKC->ERBB4->EGF->SRC->STAT3->IL2->THEMIS	214	0.00675
SAV1	RPRM	1.66E-06	8.44E-05	-0.343733166	SAV1->RASSF1->HRAS->PIK3CA->PTEN->TP53->RPRM	129	0.0068
PGRMC1	CD69	4.24E-06	0.000179068	-0.330828944	PGRMC1->M6PR->PLIN3->IGF2R->PLAUR->PLAU->JUN->IL2->CD69	101	0.00685
PITX1	LTF	6.22E-06	0.000244543	-0.325340954	PITX1->LHB->LHCGR->ARRB2->MAPK1->GRB2->ERBB2->CTNNB1->PSEN1->APP->A2M->LYZ->LTF	96	0.00685
ANGPTL4	MMD	5.51E-06	0.000221918	0.327101039	ANGPTL4->LPL->APOE->APP->PSEN1->CTNNB1->CCND1->CDK2->CDC6->MCM2->MUT->MMD	104	0.00695
CYP4F2	ACCN2	8.99E-06	0.000324246	0.319994328	CYP4F2->PTGS2->IL1B->NFKB1->PRKCA->PICK1->ACCN2	94	0.00705
IGHD	TOX2	1.68E-07	1.26E-05	0.373177976	IGHD->GHRH->POMC->NR4A1->ZNF461->TOX2	238	0.00705
PGRMC1	FCGR3B	1.78E-06	8.97E-05	-0.342797047	PGRMC1->M6PR->IGF1->IRS1->GRB2->VAV1->SYK->FCER1G->FCGR3B	133	0.0071
PSMB9	VNN2	5.40E-07	3.40E-05	0.358566101	PSMB9->PSMB7->UCHL5->PSMD4->UBC->EGFR->STAT3->IL6->ICAM1->ITGB2->VNN2	167	0.00715
PTGER1	LTF	1.09E-05	0.000379232	-0.317114186	PTGER1->BDKRB2->KNG1->KLKB1->A2M->LYZ->LTF	93	0.0072
LEPRE1	CYP2J2	1.55E-05	0.000508928	-0.311867916	LEPRE1->PPIB->BSG->MMP1->JUN->MAPK8->TP53->KAT5->PLA2G4A->CYP2J2	86	0.0072
PQBP1	MID1	3.83E-06	0.000163927	0.33226614	PQBP1->TXNL4A->CD2BP2->CD2->LCK->IL2->STAT3->EP300->SP1->SMAD3->RUNX2->SP7->MID1	109	0.00725
CHRNA3	ATP6V1E2	1.83E-06	9.14E-05	-0.342459824	CHRNA3->PSMA4->UCHL5->PSMD4->UBC->RIPK1->TRAF2->TNFRSF11A->TNFSF11->TCIRG1->ATP6V1B1->ATP6V1D->ATP6V1A->ATP6V1E2	139	0.0073
PTGER3	PTPRH	6.31E-06	0.000247045	-0.325139129	PTGER3->KNG1->EGF->GRB2->MAPK1->ELK1->SRF->PTPRH	107	0.0074
RNF43	APBB1IP	1.05E-06	5.84E-05	-0.349850518	RNF43->AKAP8L->DHX9->SF3A2->CD2BP2->CD2->LCK->LCP2->GRB2->PTK2->TLN1->APBB1IP	157	0.0074
PITX1	CRIM1	1.00E-06	5.62E-05	-0.350477646	PITX1->LHB->LHCGR->ARRB2->MAPK1->JUN->SMAD4->SMAD1->BMPR1A->BMP4->CRIM1	156	0.0074
UBQLN2	MID1	1.33E-06	7.06E-05	0.346781172	UBQLN2->PSMD4->UBC->TP53->SP1->SMAD3->RUNX2->SP7->MID1	152	0.00745
ANGPTL4	SLA	1.11E-06	6.09E-05	0.349179838	ANGPTL4->LPL->APOE->APP->PSEN1->CTNNB1->TCF4->TAL1->LMO2->LDB1->LHX3->IFT172->SLA	160	0.0075
RNF43	MYEF2	1.42E-06	7.46E-05	0.345839583	RNF43->AKAP8L->DHX9->SF3A2->YBX1->TP53->HDAC1->SIN3A->HDAC9->MYEF2	150	0.0075
COL8A1	LPHN2	1.91E-06	9.36E-05	0.34189212	COL8A1->COL8A2->ZEB1->CDH1->CTNNB1->SMAD7->TGFBR1->FKBP1A->RYR1->HOMER1->SHANK2->LPHN2	142	0.00755
SLC22A18	SNAPIN	2.29E-05	0.000687347	-0.305939789	SLC22A18->CDKN1C->CDK4->RB1->TP53->PTEN->SLC9A3R1->CFTR->STX1A->SNAP25->SNAPIN	82	0.0076
RNF43	TFCP2	3.44E-06	0.00015147	0.333744534	RNF43->AKAP8L->DHX9->SF3A2->YBX1->TP53->YY1->TFCP2	117	0.0076
IGLV4-60	HLA-DRA	1.89E-06	9.30E-05	0.342044612	IGLV4-60->C1S->SERPING1->PLG->SERPINE1->PLAU->FOS->CREB1->CIITA->HLA-DRA	144	0.00765
IGHD	VNN2	3.61E-07	2.45E-05	0.363691974	IGHD->GHRH->GCG->LEP->STAT3->IL6->ICAM1->ITGB2->VNN2	201	0.00765
SLC2A3	LPHN2	2.10E-07	1.53E-05	0.370462482	SLC2A3->SP1->SMAD3->TGFBR1->FKBP1A->RYR1->HOMER1->SHANK2->LPHN2	259	0.0077
CASP1	VNN2	8.97E-07	5.13E-05	0.351964601	CASP1->IL18->IL1B->NFKB1->ICAM1->ITGB2->VNN2	166	0.0077
SATB2	KLRD1	8.98E-06	0.00032424	-0.320011549	SATB2->HOXA2->CD1D->B2M->CD8A->LCK->SYK->TYROBP->KLRD1	104	0.0077
SLC2A3	NUPR1	1.85E-07	1.38E-05	0.371994379	SLC2A3->SP1->TP53->NUPR1	263	0.00775
SLC2A3	SNAPIN	3.53E-07	2.41E-05	0.363981086	SLC2A3->SP1->TP53->PTEN->SLC9A3R1->CFTR->STX1A->SNAP25->SNAPIN	210	0.0078
ANGPTL4	LMBR1L	1.34E-05	0.000450004	0.314072817	ANGPTL4->LPL->APOE->APP->A2M->LYZ->LCN1->LMBR1L	96	0.008
SERPINB2	SLC25A11	5.87E-06	0.000233177	0.3261711	SERPINB2->PLAU->JUN->MAPK8->TP53->BCL2->BNIP3->BCKDHB->DLD->OGDH->SLC25A11	113	0.00805
CRB3	DYNC1I2	1.90E-05	0.000594857	-0.308794323	CRB3->PARD6A->CDC42->IQGAP1->CLIP1->DCTN1-	89	0.0081

					>DCTN2->DYNC1I2		
RNF43	PIK3CG	4.89E-06	0.000200576	-0.3288067	RNF43->AKAP8L->DHX9->SF3A2->CD2BP2->CD2->LCK->LCP2->VAV1->RAC1->AKT1->PIK3CG	117	0.0081
TET3	FPR1	8.25E-07	4.81E-05	-0.353065607	TET3->NANOG->KLF4->CTNNB1->GSK3B->TP53->NPM1->NCL->S100A11->ANXA1->FPR1	177	0.00815
NFIB	NCOA7	1.67E-05	0.000536682	0.310702013	NFIB->HNF1A->HNF4A->SMAD4->SMAD3->SP1->ESR1->NCOA7	92	0.00815
APOBEC1	CCL18	1.92E-05	0.00059831	-0.30864464	APOBEC1->APOB->LDLR->APOE->APP->PSEN1->CTNNB1->BTRC->NFKBIA->NFKB1->CCL5->CCR3->CCL18	90	0.0082
ANGPTL4	MID1	5.77E-06	0.00023006	-0.326419119	ANGPTL4->LPL->APOE->APP->PSEN1->CTNNB1->MYC->SP1->SMAD3->RUNX2->SP7->MID1	114	0.00825
CXCL14	MARCKS	2.43E-06	0.000115125	0.338561971	CXCL14->CXCR4->CD4->LCK->LCP2->GRB2->WASL->CDC42->PLD1->PRKCA->MARCKS	149	0.00825
MAP7D1	HAS2	2.97E-07	2.09E-05	0.366116774	MAP7D1->ARTN->GFRA1->RET->SHC1->EGFR->STAT3->EP300->SMAD2->SMAD4->SMAD1->BMPR1A->BMP2->HAS2	248	0.00825
SAV1	MID1	3.46E-06	0.000151981	-0.333677718	SAV1->RASSF1->HRAS->RAF1->HSP90AA1->HIF1A->SP1->SMAD3->RUNX2->SP7->MID1	133	0.0083
TET3	FCGR3B	7.14E-07	4.26E-05	-0.354951043	TET3->NANOG->KLF4->CTNNB1->ERBB2->EGFR->CBL->SYK->FCER1G->FCGR3B	191	0.00835
UAP1L1	A1CF	3.12E-07	2.18E-05	-0.365504754	UAP1L1->PGM3->GLO1->AKR1B1->DECR1->NCF2->RAC1->CTNNB1->PSEN1->APP->APOE->LDLR->APOB->APOBEC1->A1CF	250	0.00835
RB1CC1	CTSA	2.18E-05	0.000662507	0.306671714	RB1CC1->PTK2->GRB2->MAPK1->MAP2K1->RAF1->YWHAZ->GP1BA->F2->GLA->GLB1->CTSA	89	0.0084
PTGER1	CGGBP1	2.80E-06	0.000127693	0.336645879	PTGER1->BDKRB2->KNG1->EGF->EGFR->STAT3->RAC1->NCKAP1->CYFIP1->FMR1->CGGBP1	143	0.0084
SERPINB2	ST3GAL1	2.68E-06	0.000123488	0.337224585	SERPINB2->PLAU->JUN->MAPK8->TP53->YBX1->SF3A2->SNRPE->LSM4->EXOSC10->CHPF->B4GALNT1->ST8SIA1->ST3GAL1	149	0.00845
SERPINB2	ENPP3	1.26E-05	0.000426426	-0.315007984	SERPINB2->PLAU->FOS->CSF2->CSF2RB->IL3->ENPP3	105	0.00845
PGRMC1	GFPT1	3.34E-06	0.000147893	0.33417722	PGRMC1->M6PR->PLIN3->IGF2R->PLAUR->PLAU->JUN->ATF3->ATF4->ASNS->GLUL->GFPT1	140	0.0086
SATB2	SHANK2	2.07E-05	0.000633637	0.307492306	SATB2->HOXA2->CD1D->B2M->CD8A->IL2->STAT3->EP300->SMAD2->TGFBR1->FKBP1A->RYR1->HOMER1->SHANK2	93	0.00865
EEF1B2	MTMR3	8.93E-07	5.11E-05	-0.35203409	EEF1B2->RPL6->RPL5->MDM2->TP53->PTEN->INPP4B->MTMR3	193	0.0087
RPS2	CRIM1	1.43E-05	0.000474091	-0.313128764	RPS2->RPS3->RPL5->MDM2->TP53->SP1->SMAD3->SMAD4->SMAD1->BMPR1A->BMP4->CRIM1	106	0.0087
CASP1	SLA	5.77E-06	0.00023006	0.326432764	CASP1->IL18->IL1B->IL8->RELA->HDAC1->TAL1->LMO2->LDB1->LHX3->IFT172->SLA	125	0.0087
IGLV4-60	CD2	6.04E-06	0.000238992	0.325780519	IGLV4-60->C1S->SERPING1->PLG->FN1->ITGA4->PXN->PTK2->GRB2->LCP2->LCK->CD2	127	0.00875
SERPINB2	SCML1	4.95E-06	0.000203068	-0.328610097	SERPINB2->PLAU->NFKB1->RELA->HDAC1->SIN3A->MECP2->CDKL5->NHS->SCML1	132	0.0088
MAP7D1	HLA-DRA	5.42E-06	0.000219116	0.327330623	MAP7D1->ARTN->GFRA1->RET->SHC1->EGFR->STAT3->EP300->CREB1->CIITA->HLA-DRA	131	0.00885
IGJ	THEMIS	4.66E-07	3.02E-05	0.360452963	IGJ->IL2->THEMIS	241	0.00885
SCEL	TAX1BP3	5.25E-07	3.32E-05	0.358918935	SCEL->TXNIP->TXN->MAP3K5->TRAF2->CHUK->RELA->EP300->STAT3->RAC1->ARHGDIA->RHOA->RTKN->TAX1BP3	229	0.0089
IGJ	TOX2	3.86E-07	2.57E-05	0.362851862	IGJ->IL2->STAT3->EP300->TP53->BCL2->NR4A1->ZNF461->TOX2	261	0.0089
FCRL5	TNFRSF18	2.40E-05	0.000716063	0.30515857	FCRL5->BCL9->CTNNB1->CCND1->STAT3->IL2->TNFRSF18	95	0.00895
EBI3	SLA	8.06E-06	0.000298255	0.321594297	EBI3->IL27RA->STAT1->EP300->MYOD1->TCF3->TAL1->LMO2->LDB1->LHX3->IFT172->SLA	124	0.00905
MAGT1	SCML1	3.53E-07	2.41E-05	0.363954356	MAGT1->DDOST->SSR4->RPS5->RPL5->MDM2->RB1->HDAC1->SIN3A->MECP2->CDKL5->NHS->SCML1	276	0.00905
SLC2A3	UPP1	6.21E-07	3.79E-05	0.35675818	SLC2A3->SP1->TP53->RRM2B->RRM1->RRM2->TYMS->TK1->CDA->TYMP->UPP1	228	0.0091
CHRNA3	CRIM1	5.31E-06	0.000215753	0.327615152	CHRNA3->PSMA4->UCHL5->PSMD4->UBC->HIF1A->EP300-	137	0.0091

					>SMAD2->SMAD4->SMAD1->BMPR1A->BMP4->CRIM1		
EEF1B2	LTF	2.75E-05	0.000797609	-0.303057591	EEF1B2->RPL6->RPL5->MDM2->TP53->GSK3B->CTNNB1->PSEN1->APP->A2M->LYZ->LTF	93	0.0092
UAP1L1	MID1	3.80E-07	2.55E-05	-0.363024241	UAP1L1->PGM3->GLO1->AKR1B1->DECR1->NCF2->RAC1->AKT1->ESR1->SP1->SMAD3->RUNX2->SP7->MID1	288	0.0092
UAP1L1	KLRD1	5.73E-07	3.55E-05	0.357802068	UAP1L1->PGM3->GLO1->AKR1B1->DECR1->NCF2->RAC1->VAV1->SYK->TYROBP->KLRD1	245	0.0092
CEACAM1	SLA	6.24E-06	0.000244812	-0.325300362	CEACAM1->PTPN11->GRB2->ERBB2->CTNNB1->TCF4->TAL1->LMO2->LDB1->LHX3->IFT172->SLA	133	0.00925
SATB2	FPR1	1.23E-05	0.000419116	-0.315331235	SATB2->HOXA2->CD1D->B2M->CD8A->IL2->STAT3->EP300->TP53->NPM1->NCL->S100A11->ANXA1->FPR1	113	0.00925
SLC2A3	GNA11	1.16E-06	6.31E-05	-0.348573118	SLC2A3->SP1->EP300->JUN->EDN1->EDNRB->GNA11	209	0.0093
CXCL14	SHANK2	3.57E-06	0.000155049	0.333221948	CXCL14->CXCR4->ITCH->RNF11->SMURF2->SMAD2->TGFBR1->FKBP1A->RYR1->HOMER1->SHANK2	160	0.00945
IGLV4-60	PIK3CG	8.37E-06	0.000307709	0.321042564	IGLV4-60->C1S->SERPING1->PLG->FN1->ITGA4->PXN->ILK->AKT1->PIK3CG	130	0.00945
SLC11A1	ST3GAL1	3.76E-06	0.000161644	0.332521107	SLC11A1->ZBTB17->MYC->EP300->TP53->YBX1->SF3A2->SNRPE->LSM4->EXOSC10->CHPF->B4GALNT1->ST8SIA1->ST3GAL1	152	0.00955
GAS1	CLEC4E	4.93E-07	3.16E-05	0.359723428	GAS1->SHH->GLI2->BTRC->NFKBIA->IKBKB->BCL10->CARD9->CLEC4E	273	0.00955
ZC3H12A	DYNC1I2	3.42E-05	0.000948334	-0.299633206	ZC3H12A->CCL2->IL8->RELA->EP300->TP53->CHEK1->CDC25C->PLK1->NUDC->PAFAH1B1->DCTN1->DCTN2->DYNC1I2	95	0.00955
SOX11	CCL18	1.44E-06	7.51E-05	0.345720175	SOX11->BNIP3->BCL2->TP53->HDAC1->RELA->NFKB1->CCL5->CCR3->CCL18	202	0.0096
OXA1L	SLC25A11	2.06E-06	9.96E-05	0.340871216	OXA1L->BCL2->BNIP3->BCKDHB->DLD->OGDH->SLC25A11	180	0.00965
MAVS	VNN2	2.96E-06	0.000134079	-0.335862145	MAVS->IRF3->CREBBP->RELA->NFKB1->ICAM1->ITGB2->VNN2	167	0.00965
IGJ	SPINK5	8.56E-07	4.93E-05	0.352590004	IGJ->IL2->STAT3->EGFR->PTPN1->CDH2->JUP->DSG1->KLK5->SPINK5	239	0.00975
SAV1	PIK3AP1	1.41E-05	0.000470655	0.313287011	SAV1->RASSF1->HRAS->PIK3CA->PIK3R1->PIK3AP1	114	0.0098
RNF43	DYNC1I2	2.58E-06	0.000120616	0.337753757	RNF43->AKAP8L->DHX9->SF3A2->YBX1->TP53->CHEK2->CDC25C->PLK1->NUDC->PAFAH1B1->DCTN1->DCTN2->DYNC1I2	175	0.00985
CHST2	PIK3CG	6.39E-07	3.88E-05	0.356393217	CHST2->SELL->CD34->MYB->CREBBP->CREB1->AKT1->PIK3CG	260	0.0099
PITX1	GRSF1	9.56E-06	0.000340026	0.319093149	PITX1->LHB->LHCGR->ARRB2->MAPK1->FOS->PTGS2->ALOX5->GPX4->GRSF1	133	0.00995
TET3	KCNQ2	2.85E-06	0.000129474	-0.336387206	TET3->NANOG->KLF4->CTNNB1->IQGAP1->CALM1->KCNQ2	174	0.01
NOP10	SLA	1.19E-05	0.000408111	0.315809627	NOP10->DKC1->RUVBL1->CTNNB1->TCF4->TAL1->LMO2->LDB1->LHX3->IFT172->SLA	126	0.01
CXCL14	BCL11A	1.56E-06	8.02E-05	0.34462473	CXCL14->CXCR4->CD4->IFNG->IL2->STAT3->SRC->ESR1->NCOA2->BCL11A	209	0.01
CEACAM1	CRIM1	1.71E-05	0.000544834	0.310426382	CEACAM1->PTPN11->JAK2->STAT3->EP300->SMAD2->SMAD4->SMAD1->BMPR1A->BMP4->CRIM1	112	0.01005
CRB3	GRSF1	2.90E-05	0.000834534	0.302234537	CRB3->PARD6A->CDC42->IQGAP1->RAC1->STAT3->IL6->PTGS2->ALOX5->GPX4->GRSF1	102	0.0101
MAP7D1	SHANK2	1.23E-05	0.000417408	-0.315408476	MAP7D1->ARTN->GFRA1->RET->GRB2->VAV1->RAC1->AKT1->MTOR->FKBP1A->RYR1->HOMER1->SHANK2	128	0.0101
COL8A1	ZFHX3	2.07E-05	0.000634868	0.307434746	COL8A1->COL8A2->ZEB1->CDH1->CTNNB1->MYC->MYB->ZFHX3	110	0.0101
SATB2	FCGR3B	1.42E-05	0.00047124	-0.313235547	SATB2->HOXA2->CD1D->B2M->CD8A->LCK->SYK->FCER1G->FCGR3B	124	0.0103
GAS1	VNN2	3.42E-06	0.00015102	0.333825491	GAS1->SHH->FGF8->FGFR1->FRS2->PTPN11->IL6ST->IL6->ICAM1->ITGB2->VNN2	173	0.01035
RNF43	ARHGEF19	8.49E-06	0.000310851	0.320834529	RNF43->AKAP8L->DHX9->SF3A2->CD2BP2->CD2->LCK->LCP2->GRB2->ERBB2->CTNNB1->DVL1->DAAM1->ARHGEF19	142	0.01035
SATB2	CCL18	8.76E-06	0.000318939	-0.3203726	SATB2->HOXA2->CD1D->B2M->CD8A->CD4->CCR5->CCL13->CCR3->CCL18	140	0.01035

PGRMC1	A1CF	1.61E-05	0.000523368	0.311270698	PGRMC1->M6PR->IGF1->IRS1->GRB2->ERBB2->CTNNB1->PSEN1->APP->APOE->LDLR->APOB->APOBEC1->A1CF	117	0.0104
SLC11A1	VNN2	3.48E-06	0.000152609	0.333580729	SLC11A1->ZBTB17->MYC->EP300->RELA->NFKB1->ICAM1->ITGB2->VNN2	171	0.0104
RNF43	SLC25A11	1.76E-06	8.89E-05	-0.342962611	RNF43->AKAP8L->DHX9->SF3A2->YBX1->TP53->BCL2->BNIP3->BCKDHB->DLD->OGDH->SLC25A11	215	0.0104
HLA-DQB2	HLA-DRA	1.53E-05	0.000503985	0.312031317	HLA-DQB2->ZFYVE9->SMAD2->EP300->CREB1->CIITA->HLA-DRA	123	0.01045
UBXN7	CD2	2.02E-06	9.81E-05	-0.341135279	UBXN7->CUL2->RBX1->CUL1->BTRC->NFKBIA->LCK->CD2	210	0.0105
DDX47	MARCKS	7.59E-07	4.49E-05	-0.354149614	DDX47->DNAH8->VCP->ATXN3->UBC->EGFR->GRB2->WASL->CDC42->PLD1->PRKCA->MARCKS	291	0.01055
RB1CC1	C18orf10	6.07E-06	0.000239818	-0.325694458	RB1CC1->PTK2->SRC->ESR1->EP300->RELA->NFKB1->C18orf10	161	0.0106
NOP10	CRIM1	3.55E-05	0.000974527	-0.299056053	NOP10->DKC1->RUVBL1->CTNNB1->SMAD4->SMAD1->BMPR1A->BMP4->CRIM1	107	0.0106
RNF43	VPS4B	1.31E-05	0.000441317	-0.314413427	RNF43->AKAP8L->DHX9->SF3A2->YBX1->TP53->UBC->HGS->VPS4A->CHMP2A->VPS4B	133	0.01065
PGRMC1	MID1	7.62E-06	0.000287177	0.322407365	PGRMC1->M6PR->IGF1->IGF1R->ESR1->SP1->SMAD3->RUNX2->SP7->MID1	153	0.0107
MAGT1	HMOX1	6.70E-06	0.00025921	-0.324284042	MAGT1->DDOST->SSR4->RPS5->RPL5->MDM2->TP53->HIF1A->HMOX1	157	0.0107
UAP1L1	SHANK2	1.58E-06	8.07E-05	-0.344463882	UAP1L1->PGM3->GLO1->AKR1B1->DECR1->NCF2->RAC1->AKT1->MTOR->FKBP1A->RYR1->HOMER1->SHANK2	232	0.0107
CXCL14	CTSA	2.58E-06	0.000120616	0.33773377	CXCL14->CXCR4->CD4->CD8A->B2M->CD1D->CTSA	202	0.01075
SLC2A3	SOCS7	1.85E-06	9.17E-05	-0.342321349	SLC2A3->SP1->ESR1->SRC->VCL->SORBS3->SOCS7	230	0.01095
SATB2	UPP1	1.95E-05	0.000609315	-0.308350407	SATB2->HOXA2->CD1D->B2M->CD8A->IL2->STAT3->EP300->TP53->RRM2B->RRM1->RRM2->TYMS->TK1->CDA->TYMP->UPP1	119	0.01095
SOX11	SMAD7	1.43E-05	0.000475369	0.313072249	SOX11->BNIP3->BCL2->TP53->EP300->SMAD2->TGFBR1->SMAD7	134	0.01105
CRB3	ZMYM4	3.73E-06	0.000160668	-0.332625532	CRB3->PARD6A->CDC42->WASL->GRB2->FRS2->FGFR1->ZMYM4	179	0.01105
MAP7D1	CYP2J2	4.18E-06	0.000176829	-0.331025812	MAP7D1->ARTN->GFRA1->RET->SHC1->EGFR->UBC->TP53->KAT5->PLA2G4A->CYP2J2	172	0.01105
PGRMC1	SLC25A11	3.47E-06	0.000152238	-0.333634421	PGRMC1->M6PR->IGF1->IRS1->MAPK8->TP53->BCL2->BNIP3->BCKDHB->DLD->OGDH->SLC25A11	189	0.0111
MBNL2	MED23	1.20E-05	0.000409891	0.315728469	MBNL2->CCT3->PPP2CA->SGOL1->PLK1->CDC25C->CHEK2->BRCA1->ESR1->NCOA2->VDR->MED1->CDK8->MED23	140	0.0111
SLC2A3	MID1	1.40E-06	7.37E-05	-0.346042473	SLC2A3->SP1->SMAD3->RUNX2->SP7->MID1	254	0.0112
RABGEF1	ST3GAL1	9.85E-06	0.000349046	-0.318640178	RABGEF1->RABEP1->RAB5A->EGFR->GRB2->LCP2->LCK->CD2->CD2BP2->SF3A2->SNRPE->LSM4->EXOSC10->CHPF->B4GALNT1->ST8SIA1->ST3GAL1	152	0.01125
CHRNA3	RUNDC3A	1.28E-05	0.000431672	0.314792733	CHRNA3->PSMA4->UCHL5->PSMD4->UBC->EGFR->NCK1->TNIK->RAP2A->RUNDC3A	140	0.01125
SLC22A18	SLA	5.43E-06	0.000219116	-0.327309623	SLC22A18->CDKN1C->MYOD1->TCF3->TAL1->LMO2->LDB1->LHX3->IFT172->SLA	174	0.0113
MBNL2	PREPL	3.33E-06	0.000147688	0.334216387	MBNL2->CCT3->PPP2CA->SGOL1->PLK1->CDC25C->CHEK2->TP53->HDAC1->RELA->IKBKB->PPM1B->PREPL	197	0.01135
CD79A	VNN2	7.27E-06	0.000276801	0.323083366	CD79A->BLNK->GRB2->EGFR->STAT3->IL6->ICAM1->ITGB2->VNN2	166	0.0114
UBQLN2	SCML1	3.09E-06	0.000138906	0.335250114	UBQLN2->PSMD4->UBC->TP53->HDAC1->SIN3A->MECP2->CDKL5->NHS->SCML1	206	0.01145
IGLV4-60	CD69	1.32E-05	0.00044404	0.314305129	IGLV4-60->C1S->SERPING1->PLG->SERPINE1->PLAU->JUN->IL2->CD69	142	0.01145
ANGPTL4	ST3GAL1	4.71E-06	0.000195337	0.329312252	ANGPTL4->LPL->APOE->APP->PSEN1->CTNNB1->GSK3B->TP53->YBX1->SF3A2->SNRPE->LSM4->EXOSC10->CHPF->B4GALNT1->ST8SIA1->ST3GAL1	185	0.0116
CXCL14	GALNT1	4.80E-06	0.000197895	-0.329052154	CXCL14->CXCR4->CD4->LCK->CD2->CD2BP2->SF3A2->SNRPD3->LSM7->EXOSC6->CHPF->GALNT1	185	0.0116
FCRL5	APBB1IP	1.61E-05	0.000523368	0.311289771	FCRL5->BCL9->CTNNB1->SRC->PTK2->TLN1->APBB1IP	134	0.01165
MAP7D1	SMAD7	3.13E-05	0.000880031	0.30105882	MAP7D1->ARTN->GFRA1->RET->GRB2->ERBB2->CTNNB1-	113	0.01165

					>SMAD7		
RNF43	FCGR3B	9.87E-06	0.000349199	-0.318616991	RNF43->AKAP8L->DHX9->SF3A2->CD2BP2->CD2->LCK->SYK->FCER1G->FCGR3B	158	0.01165
SATB2	VNN2	2.39E-06	0.000113505	-0.338777614	SATB2->HOXA2->CD1D->B2M->CD8A->IL2->STAT3->IL6->ICAM1->ITGB2->VNN2	235	0.01165
RNF43	CTSA	7.02E-06	0.00026876	0.323599726	RNF43->AKAP8L->DHX9->SF3A2->CD2BP2->CD2->LCK->CD8A->B2M->CD1D->CTSA	170	0.0117
SLC22A18	MMD	2.73E-05	0.000792661	-0.303183671	SLC22A18->CDKN1C->CDK4->PCNA->CDT1->CDC6->MCM2->MUT->MMD	118	0.0118
RNF43	ASS1	2.24E-05	0.000677548	-0.306235905	RNF43->AKAP8L->DHX9->SF3A2->CD2BP2->CD2->LCK->LCP2->VAV1->RAC1->AKT1->NOS3->ARG2->OTC->ASS1	127	0.0118
PTGER3	NUPR1	1.90E-05	0.000594744	0.308812573	PTGER3->KNG1->EGF->EGFR->UBC->TP53->NUPR1	133	0.0119
CCL7	CLEC4E	1.82E-06	9.11E-05	0.34256347	CCL7->CCR1->CCL5->NFKB1->IKBKB->BCL10->CARD9->CLEC4E	266	0.01195
MMP8	FPR1	1.91E-05	0.00059831	0.308670801	MMP8->TIMP2->MMP2->JUN->MAPK8->TP53->NPM1->NCL->S100A11->ANXA1->FPR1	134	0.01205
EEF1B2	CTSA	3.65E-05	0.000993534	-0.298597401	EEF1B2->RPL6->RPL5->MDM2->AKT1->TSC2->YWHAZ->GP1BA->F2->GLA->GLB1->CTSA	114	0.0121
TET3	GFPT1	4.52E-06	0.000189935	0.329899569	TET3->NANOG->KLF4->CTNNB1->GSK3B->TP53->ATF3->ATF4->ASNS->GLUL->GFPT1	198	0.0121
CHST2	GATA3	2.03E-06	9.86E-05	0.341034993	CHST2->SELL->CD34->MYB->CREBBP->TP53->EP300->STAT3->IL2->IL5->GATA3	260	0.01215
MAP7D1	GATA3	3.59E-05	0.00098161	0.29885108	MAP7D1->ARTN->GFRA1->RET->SHC1->IL2->IL5->GATA3	116	0.01215
IGKC	SLA	1.52E-05	0.000499379	0.312185892	IGKC->ERBB4->EGF->SRC->ESR1->BRCA1->RBBP8->LMO4->LDB1->LHX3->IFT172->SLA	148	0.01225
RNF4	SLC25A3	2.60E-05	0.000763522	0.303956523	RNF4->AR->NCOA3->EP300->TP53->APAF1->CYCS->CYC1->UQCRFS1->SLC25A3	127	0.01235
SLC2A3	ESRRG	4.29E-06	0.000180974	-0.330660142	SLC2A3->SP1->TP53->SIRT1->PPARGC1A->ESRRG	210	0.01255
MMP8	ESRRG	3.42E-05	0.000948334	-0.299626799	MMP8->TIMP2->MMP2->JUN->MAPK8->TP53->SIRT1->PPARGC1A->ESRRG	122	0.01265
JHDM1D	SOS2	3.07E-06	0.000138454	0.335335458	JHDM1D->MBD2->HDAC1->TP53->UBC->EGFR->GRB2->SOS2	248	0.0127
PACS2	GCLC	8.47E-06	0.000310627	0.320862266	PACS2->CANX->CFTR->SLC9A3R1->PDGFRB->PIK3R2->IRS1->MAPK8->GSTP1->GSS->GCLC	184	0.0128
PTGER1	CYP2J2	3.06E-05	0.000869099	-0.301383122	PTGER1->BDKRB2->KNG1->EGF->EGFR->UBC->TP53->KAT5->PLA2G4A->CYP2J2	126	0.01285
MAP7D1	ATP6V1E1	6.97E-06	0.000267263	0.323698451	MAP7D1->ARTN->GFRA1->RET->SHC1->EGFR->UBC->RIPK1->TRAF2->TNFRSF11A->TNFSF11->TCIRG1->ATP6V1B1->ATP6V1D->ATP6V1A->ATP6V1E1	202	0.01305
SAV1	CRYZ	3.66E-05	0.000995043	0.298545733	SAV1->RASSF1->HRAS->PIK3CA->AKT1->RAC1->NCF2->DECR1->CRYZ	128	0.0132
UAP1L1	HMOX1	5.99E-06	0.000237453	0.325891643	UAP1L1->PGM3->GLO1->AKR1B1->DECR1->NCF2->RAC1->AKT1->HIF1A->HMOX1	223	0.0132
PARVA	MTMR3	2.38E-05	0.000710912	0.305285146	PARVA->ILK->PXN->PTK2->PTEN->INPP4B->MTMR3	144	0.01345
CXCL14	RBM39	1.25E-05	0.00042623	0.315051436	CXCL14->CXCR4->CD4->LCK->CD2->CD2BP2->SF3A2->RBM39	177	0.0136
SLC22A18	CGGBP1	3.25E-05	0.000905063	-0.300448107	SLC22A18->CDKN1C->CDK4->CDKN1B->AKT1->RAC1->NCKAP1->CYFIP1->FMR1->CGGBP1	135	0.01365
EEF1B2	CRIM1	2.05E-05	0.00063198	-0.307577752	EEF1B2->RPL6->RPL5->MDM2->TP53->SP1->SMAD3->SMAD4->SMAD1->BMPR1A->BMP4->CRIM1	152	0.0137
IGLV4-60	CXCL13	3.05E-05	0.000867406	0.301442273	IGLV4-60->C1S->SERPING1->PLG->FN1->ITGA4->PXN->PTK2->GRB2->LCP2->LCK->CD4->CXCR4->CXCL13	137	0.01375
RNF43	ESRRG	3.57E-05	0.000977869	0.298960131	RNF43->AKAP8L->DHX9->SF3A2->YBX1->TP53->SIRT1->PPARGC1A->ESRRG	134	0.01385
SLC2A3	BCL11A	4.54E-06	0.000190213	-0.329840798	SLC2A3->SP1->ESR1->NCOA2->BCL11A	250	0.014
SLC2A3	SHANK2	9.33E-06	0.000333629	-0.319439964	SLC2A3->SP1->SMAD3->TGFBR1->FKBP1A->RYR1->HOMER1->SHANK2	203	0.014
LEPRE1	SLA	2.71E-05	0.00078953	0.303303481	LEPRE1->PPIB->BSG->MMP1->JUN->MAPK8->TP53->BRCA1->RBBP8->LMO4->LDB1->LHX3->IFT172->SLA	144	0.014
CDON	HAS2	2.69E-05	0.000786562	0.30342045	CDON->SHH->NOG->BMP2->HAS2	143	0.014

MAP7D1	VNN2	3.53E-06	0.000153911	0.333394025	MAP7D1->ARTN->GFRA1->RET->SHC1->EGFR->STAT3->IL6->ICAM1->ITGB2->VNN2	271	0.01405
ADAMTS16	LPHN2	9.01E-06	0.000324573	0.319962598	ADAMTS16->EGR1->FOS->JUN->EP300->SMAD2->TGFBR1->FKBP1A->RYR1->HOMER1->SHANK2->LPHN2	206	0.0141
RB1CC1	SCML1	2.51E-05	0.000741594	0.304512115	RB1CC1->PTK2->PTEN->TP53->HDAC1->SIN3A->MECP2->CDKL5->NHS->SCML1	153	0.0142
UAP1L1	SNAPIN	6.39E-06	0.000248793	0.324961151	UAP1L1->PGM3->GNPNAT1->HK2->SLC2A4->STX4->SNAP25->SNAPIN	236	0.0142
CXCL14	RNF135	8.92E-06	0.000322994	0.320102052	CXCL14->CXCR4->CD4->IFNG->IL18->IL1B->MYD88->IRF7->TBK1->IRF3->MAVS->DDX58->RNF135	213	0.0143
SATB2	SUV420H1	1.51E-05	0.000496952	0.312307154	SATB2->HOXA2->CD1D->B2M->CD8A->IL2->STAT3->EP300->ESR1->NCOA2->SUV420H1	178	0.01435
UBXN7	FCGR3B	4.56E-06	0.000190696	-0.329785835	UBXN7->CUL2->VHL->HIF1A->UBC->EGFR->CBL->SYK->FCER1G->FCGR3B	256	0.01445
CHST2	ITGA6	4.85E-06	0.00019956	-0.328914288	CHST2->SELL->CD34->MYB->CREBBP->TP53->PTEN->PTK2->PXN->ITGA4->ITGB1->ITGA6	259	0.01445
PSMB9	MTMR3	2.99E-05	0.000855046	-0.301739051	PSMB9->PSMB7->UCHL5->PSMD4->UBC->TP53->PTEN->INPP4B->MTMR3	145	0.01445
TET3	CCL18	1.00E-05	0.000354731	-0.318351538	TET3->NANOG->KLF4->CTNNB1->BTRC->NFKBIA->NFKB1->CCL5->CCR3->CCL18	210	0.0145
RNF43	CRYZ	1.67E-05	0.000534811	-0.310786181	RNF43->AKAP8L->DHX9->SF3A2->CD2BP2->CD2->LCK->LCP2->VAV1->RAC1->NCF2->DECR1->CRYZ	176	0.01475
TET3	RBM39	1.64E-05	0.000529655	0.311042587	TET3->NANOG->KLF4->CTNNB1->GSK3B->TP53->YBX1->SF3A2->RBM39	179	0.0149
RNF43	RNF135	1.74E-05	0.000551754	0.310141543	RNF43->AKAP8L->DHX9->SF3A2->YBX1->TP53->CREBBP->IRF3->MAVS->DDX58->RNF135	178	0.015
SATB2	C18orf10	1.20E-05	0.000410265	-0.315698562	SATB2->HOXA2->CD1D->B2M->CD8A->LCK->NFKBIA->NFKB1->C18orf10	208	0.015
PSMB9	CLEC2D	5.37E-06	0.000217578	0.32746267	PSMB9->PSMB7->UCHL5->PSMD4->UBC->RIPK1->TRAF2->TNFRSF11A->TNFSF11->CLEC2D	269	0.01515
CXCL14	UPP1	1.59E-05	0.000518949	-0.311477916	CXCL14->CXCR4->CD4->IFNG->IL2->STAT3->EP300->TP53->RRM2B->RRM1->RRM2->TYMS->TK1->CDA->TYMP->UPP1	187	0.01525
MAP7D1	SCML1	9.45E-06	0.000336822	-0.319249389	MAP7D1->ARTN->GFRA1->RET->SHC1->EGFR->UBC->TP53->HDAC1->SIN3A->MECP2->CDKL5->NHS->SCML1	236	0.01535
UAP1L1	BCL11A	5.70E-06	0.000227841	-0.326613138	UAP1L1->PGM3->GLO1->AKR1B1->DECR1->NCF2->RAC1->AKT1->ESR1->NCOA2->BCL11A	282	0.01555
NOP10	CLEC4E	7.59E-06	0.000286296	0.322469636	NOP10->DKC1->RUVBL1->CTNNB1->BTRC->NFKBIA->IKBKG->BCL10->CARD9->CLEC4E	269	0.016
SCEL	MMD	9.04E-06	0.000325423	-0.319907275	SCEL->TXNIP->TXN->MAP3K5->TRAF2->RIPK1->UBC->TP53->RPA1->RPA3->MCM2->MUT->MMD	259	0.0162
ANGPTL4	SCML1	3.21E-05	0.000899085	-0.300636938	ANGPTL4->LPL->APOE->APP->PSEN1->CTNNB1->GSK3B->TP53->HDAC1->SIN3A->MECP2->CDKL5->NHS->SCML1	168	0.01625
RNF43	SEMA4B	2.81E-05	0.000811673	-0.30274118	RNF43->AKAP8L->DHX9->SF3A2->CD2BP2->CD2->LCK->PTPRC->FYN->GRIN2A->DLG4->SEMA4B	172	0.0163
SLC2A3	CD2	2.28E-05	0.000687117	0.305959824	SLC2A3->SP1->EP300->STAT3->IL2->LCK->CD2	191	0.01645
TET3	ARHGEF19	3.60E-05	0.00098161	0.298830361	TET3->NANOG->KLF4->CTNNB1->DVL1->DAAM1->ARHGEF19	169	0.01665
SCEL	CD55	1.33E-05	0.000446688	0.314199866	SCEL->TXNIP->TXN->MAP3K5->TRAF2->RIPK1->UBC->EGFR->GRB2->VAV1->CD19->CR2->C3->CD55	244	0.0167
CHST2	KCNQ2	8.63E-06	0.000314975	0.320590117	CHST2->SELL->CD34->MYB->CREBBP->CREB1->CAMK2B->CALM1->KCNQ2	286	0.0169
PGRMC1	SLA	2.32E-05	0.00069471	-0.305730601	PGRMC1->M6PR->IGF1->IGF1R->ESR1->BRCA1->RBBP8->LMO4->LDB1->LHX3->IFT172->SLA	201	0.01725
UBXN7	BEX1	2.37E-05	0.000708215	-0.305358767	UBXN7->CUL2->VHL->HIF1A->UBC->EGFR->SHC1->NGFR->BEX1	212	0.018
SAV1	SCML1	3.64E-05	0.000990471	-0.298674015	SAV1->RASSF1->HRAS->PIK3CA->PTEN->TP53->HDAC1->SIN3A->MECP2->CDKL5->NHS->SCML1	187	0.018
RNF43	C18orf10	1.78E-05	0.000564015	-0.309776023	RNF43->AKAP8L->DHX9->SF3A2->CD2BP2->CD2->LCK->NFKBIA->NFKB1->C18orf10	242	0.01815
ANGPTL4	VNN2	2.64E-05	0.000774518	0.303719309	ANGPTL4->LPL->APOE->APP->PSEN1->CTNNB1->BTRC->NFKBIA->NFKB1->ICAM1->ITGB2->VNN2	204	0.01825
SLC2A3	ABCB1	2.22E-05	0.000672361	-0.306414197	SLC2A3->SP1->TP53->ABCB1	226	0.01835

PTGER1	HAS2	3.07E-05	0.000869802	0.301356166	PTGER1->BDKRB2->KNG1->EGF->SRC->ESR1->EP300->SMAD2->SMAD4->SMAD1->BMPR1A->BMP2->HAS2	202	0.01855
CHST2	SNAPIN	1.40E-05	0.000466868	0.31343989	CHST2->SELL->CD34->MYB->CREBBP->TP53->PTEN->SLC9A3R1->CFTR->STX1A->SNAP25->SNAPIN	277	0.01865
UBXN7	HMOX1	3.56E-05	0.00097482	-0.299023683	UBXN7->CUL2->VHL->HIF1A->HMOX1	208	0.0193
MMP8	KANK4	3.26E-05	0.000908505	0.300374248	MMP8->TIMP2->MMP2->JUN->MAPK8->TP53->TBP->POLR2A->POLR2H->POLR3D->POLR3F->POLR3C->KANK4	219	0.01955
UAP1L1	RBM39	2.05E-05	0.000629688	-0.307648646	UAP1L1->PGM3->GLO1->AKR1B1->DECR1->NCF2->RAC1->VAV1->LCP2->LCK->CD2->CD2BP2->SF3A2->RBM39	254	0.01955
GAS1	TOX2	3.53E-05	0.00097178	0.299149852	GAS1->SHH->FGF8->FGFR1->FRS2->GRB2->VAV1->RAC1->AKT1->NR4A1->ZNF461->TOX2	215	0.0197
EBI3	CLEC2D	1.96E-05	0.000611367	0.308283654	EBI3->IL27RA->STAT1->EP300->RELA->CHUK->TRAF2->TNFRSF11A->TNFSF11->CLEC2D	270	0.02025
PITX1	DNAJB8	3.04E-05	0.000865461	0.301491816	PITX1->LHB->LHCGR->ARRB2->MAPK1->MYC->EP300->MEF2D->HDAC4->DNAJB8	240	0.0206
UBE2CBP	SYTL3	3.48E-05	0.000962883	0.299344	UBE2CBP->UBE2C->BUB1B->CCNB1->CDC25C->CHEK1->TP53->PTEN->SLC9A3R1->CFTR->STX1A->SYTL4->RAB27A->SYTL3	240	0.021
EBI3	SLC39A2	3.02E-05	0.00086087	0.301618215	EBI3->IL27RA->STAT1->EP300->CREB1->CAMK2B->GRIN2B->DLG4->KCNA4->KCNAB2->SLC39A2	266	0.0221
APOBEC1	CLEC4E	2.83E-05	0.000817135	-0.302593386	APOBEC1->APOB->LDLR->APOE->APP->PSEN1->CTNNB1->BTRC->NFKBIA->IKBKG->BCL10->CARD9->CLEC4E	280	0.02225

Table S4 - The 473 transition genes and the number of signal propagation paths crossed it

Transition gene name	Number of signal propagation paths crossed it
TP53	139
CTNNB1	100
EP300	94
GRB2	85
STAT3	83
LCK	82
IL2	79
EGFR	74
UBC	66
SF3A2	53
APP	48
AKT1	47
CD8A	47
PSEN1	46
APOE	45
CD4	45
RAC1	44
SYK	43
SP1	41
AKAP8L	38
DHX9	38
LCP2	37
ESR1	36
ERBB2	35
JUN	34
CREB1	33
CD2	32
CD2BP2	32
CREBBP	31
SRC	31
EGF	30
NFKB1	29
PSMD4	28
RET	27
CXCR4	26
HIF1A	26
RELA	26
SHC1	26
UCHL5	26

VAV1	26
SHH	25
ARTN	24
CD1D	24
GFRA1	24
IFNG	24
LPL	24
PLAU	24
APOB	23
B2M	23
MAPK8	23
CBL	22
FKBP1A	22
HOMER1	22
HOXA2	22
LDLR	22
NFKBIA	22
PTPN11	22
RYR1	22
STAT1	22
YBX1	22
DKC1	21
HDAC1	21
KNG1	21
MYC	21
PSMB7	21
IFT172	20
NCOA2	20
PTK2	20
SMAD2	20
SMAD4	20
IL18	19
LDB1	19
LHX3	19
CCR3	18
CCR5	18
CIITA	18
FCER1G	18
FGF8	18
FGFR1	18
TRAF2	18

APOBEC1	17
MYB	17
SMAD1	17
BCL2	16
BDKRB2	16
BMPR1A	16
GSK3B	16
IRF3	16
PTEN	16
SIN3A	16
TGFBR1	16
ZBTB17	16
CDC42	15
MDM2	15
RUVBL1	15
TYROBP	15
BLNK	14
BMP4	14
BTRC	14
CALM1	14
GHRH	14
HRAS	14
ITGB2	14
KAT5	14
M6PR	14
SMAD3	14
ANXA1	13
CCL5	13
GCG	13
ICAM1	13
IL6	13
LEP	13
MAPK1	13
NCL	13
NPM1	13
PIK3R1	13
S100A11	13
CD34	12
CDA	12
CDKL5	12
DECR1	12

ERBB4	12
IL27RA	12
LMO2	12
MECP2	12
NCF2	12
NHS	12
PRKCA	12
RRM1	12
RRM2	12
RRM2B	12
SELL	12
TAL1	12
TK1	12
TYMP	12
TYMS	12
IGF1	11
IRS1	11
PLCG1	11
RASSF1	11
RPL5	11
RUNX2	11
SP7	11
BCL9	10
BNIP3	10
CDC25C	10
CFTR	10
CHUK	10
DAAM1	10
DVL1	10
IQGAP1	10
JAK2	10
MTOR	10
PLD1	10
PLK1	10
SHANK2	10
SLC9A3R1	10
TNFRSF11A	10
TNFSF11	10
BCL10	9
CARD9	9
PLA2G4A	9

SNAP25	9
STX1A	9
BRCA1	8
CAMK2B	8
CCND1	8
CHPF	8
FRS2	8
IGF1R	8
KLF4	8
NANOG	8
PGM3	8
PXN	8
RIPK1	8
YY1	8
A2M	7
AKR1B1	7
AR	7
ASNS	7
ATF3	7
ATF4	7
CDKN1C	7
CHEK2	7
GLO1	7
GLUL	7
IL1B	7
LMO4	7
PIK3CA	7
PPARGC1A	7
RBBP8	7
SIRT1	7
SOS1	7
TERT	7
ARRB2	6
BCKDHB	6
BSG	6
CCL13	6
CD79A	6
DLD	6
EDNRB	6
EXOSC10	6
ILK	6

ITGA4	6
LHB	6
LHCGR	6
LSM4	6
LYZ	6
MMP1	6
MYOD1	6
NR4A1	6
OGDH	6
PARD6A	6
PDGFRB	6
PLG	6
POLR2A	6
POLR2H	6
POLR3C	6
POLR3D	6
POLR3F	6
PPIB	6
PPP2CA	6
PTGS2	6
RAF1	6
RPL6	6
SGOL1	6
SNRPE	6
TBP	6
TLN1	6
B4GALNT1	5
C1S	5
C3	5
CD19	5
CDH2	5
CR2	5
DCTN1	5
DCTN2	5
FN1	5
FOS	5
HNF1A	5
HNF4A	5
IFNGR1	5
IKBKB	5
IKBKG	5

JUP	5
KRAS	5
PSMA4	5
SERPING1	5
ST8SIA1	5
TCF4	5
WASL	5
ARHGDIA	4
ATP6V1A	4
ATP6V1B1	4
ATP6V1D	4
BMP2	4
CDK4	4
CHEK1	4
CUL2	4
DSG1	4
F2	4
GLA	4
GLB1	4
GP1BA	4
HDAC9	4
IL3	4
IL5	4
INPP4B	4
ITGB1	4
KLK5	4
MAPK14	4
MAPKAPK2	4
MMP2	4
NCOA3	4
NGFR	4
NUDC	4
PAFAH1B1	4
PRTN3	4
RB1	4
RHOA	4
SRF	4
TCF3	4
TCIRG1	4
TIMP2	4
YWHAZ	4

ZNF461	4
ACO2	3
ARG2	3
CCT3	3
CDH1	3
COL4A3	3
COL4A4	3
CS	3
CYFIP1	3
DDOST	3
EDN1	3
ELK1	3
FH	3
FMR1	3
GDNF	3
GLI2	3
HSP90AA1	3
IGBP1	3
IGF2R	3
IL8	3
KLKB1	3
LRP6	3
LSM7	3
MAP3K5	3
MCM2	3
MDH2	3
MUT	3
NCKAP1	3
NOS3	3
OTC	3
PITX2	3
PLAUR	3
PLIN3	3
POMC	3
PTPN6	3
RAB5A	3
RABEP1	3
RPS5	3
RTKN	3
SDHB	3
SNRPD3	3

SPI1	3
SSR4	3
SUCLG1	3
TGFB1	3
TSC2	3
TXN	3
TXNIP	3
TXNL4A	3
VCL	3
VHL	3
ZFYVE9	3
ADCYAP1	2
ALOX5	2
APAF1	2
ARHGEF7	2
AXIN1	2
BNIP2	2
CCL2	2
CCR1	2
CDC6	2
CDKN1B	2
COL8A2	2
CSF2RB	2
CXCR3	2
DDX58	2
DLG4	2
EXOSC6	2
GJA1	2
GMPS	2
GPX4	2
IMPDH2	2
LCN1	2
MAVS	2
MYBPC3	2
MYH6	2
NCK1	2
NOG	2
PCNA	2
PICK1	2
PTGDS	2
RAP2A	2

RPS3	2
SERPINE1	2
TNIK	2
TNNT2	2
TPM1	2
USP7	2
VIPR1	2
ZEB1	2
ADCYAP1R1	1
APBB1	1
ATF2	1
ATXN3	1
BUB1B	1
CANX	1
CASP3	1
CCL11	1
CCNB1	1
CD28	1
CD80	1
CDH5	1
CDK2	1
CDK8	1
CDS2	1
CDT1	1
CHMP2A	1
CLIP1	1
CLSPN	1
COPB1	1
COPB2	1
CSF2	1
CTNNA1	1
CUL1	1
CXCL9	1
CYC1	1
CYCS	1
DNAH8	1
DSP	1
EGR1	1
FGF23	1
FGFR2	1
FYN	1

GADD45G	1
GALNT3	1
GCNT1	1
GIT1	1
GNB2L1	1
GNPNAT1	1
GRIN2A	1
GRIN2B	1
GSS	1
GSTP1	1
GZMB	1
HDAC4	1
HGS	1
HK2	1
IL6ST	1
IRF7	1
ITCH	1
ITGA8	1
KCNA4	1
KCNAB2	1
LSM1	1
MAP2K1	1
MAPK3	1
MBD2	1
MED1	1
MEF2D	1
MRVI1	1
MYD88	1
NTRK1	1
PAK1	1
PIK3R2	1
PPM1B	1
PPP1R12A	1
PRKCE	1
PRKG1	1
PRKG2	1
PTPN1	1
PTPRC	1
RAB27A	1
RBBP4	1
RBX1	1

RFXANK	1
RNF11	1
ROCK2	1
RPA1	1
RPA3	1
SELE	1
SELPLG	1
SLC2A4	1
SMAD7	1
SMURF2	1
SORBS3	1
STX4	1
SYTL4	1
TBK1	1
TGFBI	1
TIMP1	1
UBE2C	1
UPF1	1
UPF2	1
UQCRFS1	1
VCP	1
VDR	1
VPS4A	1
ZAP70	1
